# Enhanced osteogenesis of mesenchymal stem cells encapsulated in injectable microporous hydrogel

**DOI:** 10.1038/s41598-024-65731-9

**Published:** 2024-06-25

**Authors:** Seth D. Edwards, Mrinal Ganash, Ziqiang Guan, Jeil Lee, Young Jo Kim, Kyung Jae Jeong

**Affiliations:** 1 Department of Chemical Engineering and Bioengineering, University of New Hampshire, Durham, NH 03824 USA; 2 Department of Biological and Chemical Engineering, Hongik University, Sejong City, Republic of Korea

**Keywords:** Microgel, Injectable hydrogels, Bone tissue engineering, Mesenchymal stem cell delivery, Stem cells, Health care, Materials science

## Abstract

Delivery of therapeutic stem cells to treat bone tissue damage is a promising strategy that faces many hurdles to clinical translation. Among them is the design of a delivery vehicle which promotes desired cell behavior for new bone formation. In this work, we describe the use of an injectable microporous hydrogel, made of crosslinked gelatin microgels, for the encapsulation and delivery of human mesenchymal stem cells (MSCs) and compared it to a traditional nonporous injectable hydrogel. MSCs encapsulated in the microporous hydrogel showed rapid cell spreading with direct cell–cell connections whereas the MSCs in the nonporous hydrogel were entrapped by the surrounding polymer mesh and isolated from each other. On a per-cell basis, encapsulation in microporous hydrogel induced a 4 × increase in alkaline phosphatase (ALP) activity and calcium mineral deposition in comparison to nonporous hydrogel, as measured by ALP and calcium assays, which indicates more robust osteogenic differentiation. RNA-seq confirmed the upregulation of the genes and pathways that are associated with cell spreading and cell–cell connections, as well as the osteogenesis in the microporous hydrogel. These results demonstrate that microgel-based injectable hydrogels can be useful tools for therapeutic cell delivery for bone tissue repair.

## Introduction

Existing treatments for bone defects are insufficient to meet the current need. Autograft and allograft—the current gold standard—have complications related to the scarcity of donor tissue, surgical complications, and insufficient integration of allogeneic tissue^1,2^. Injection of therapeutic cells is a promising approach to improve tissue regeneration, because it is minimally invasive, and the cells can be derived from the patient, diminishing the risk of a foreign body response. Mesenchymal stem cell (MSC) delivery has been considered as a potential treatment for bone defects because MSCs are known to promote wound healing and they readily differentiate into osteoblasts^3,4^. Despite some positive outcomes, MSC delivery for bone repair faces limitations due to several significant barriers to translation, including low cell viability and retention at the site of injection, leading to disappointing therapeutic efficacy after injection^5^.

Injectable biomaterials have been shown to improve cell retention and survival at the injection site, improving the practicality of stem cell delivery^6,7^. To promote MSC osteogenic differentiation for bone repair, the materials of the injectable hydrogel should have the potential to provide physical, mechanical, and biochemical cues to the delivered cells. Fundamental studies, mainly in 2D systems, have demonstrated that MSC differentiation is influenced by various physical properties of the environment, such as stiffness^8^, micro/nano-topography^9^, and mechanical stimulation^10^. In general, stiff substrates are known to promote osteogenic differentiation of MSCs^11^. It has recently been shown that substrate stress-relaxation^12^ and matrix degradability^13^ also play important roles in regulating MSC differentiation in 3D.

In addition to mechanical properties, cell–cell connections, mediated by N-Cadherin are known to affect MSC cell behavior. In the context of MSC osteogenic differentiation, physical contacts between cells regulate the Notch signaling pathway, which has previously been shown to positively influence osteogenic differentiation^14,15^, although conflicting results have also been reported^16^. Cadherin mediated cell–cell adhesions are involved with cell mechanotransduction, have crosstalk with focal adhesion-mediated signalling^17^, and have been shown to influence MSC osteogenesis^18^. Additionally, cells cultured in aggregate rely more on cell–cell connections rather than cell–matrix interactions to drive phenotypic changes such as stem cell differentiation^19^.

For these reasons, it is well-established that porous scaffolds enhance osteogenic differentiation of MSCs (µm length scale)^20–22^. However, most injectable hydrogels are non-porous (with the typical mesh size in the nanometer scale), and delivery of MSCs in stiff nonporous injectable hydrogels inhibits cell spreading^23^, migration^24,25^, and cell–cell communication^26,27^, all of which play important roles in regulating osteogenic differentiation of MSCs. Recently, injectable microporous hydrogels based on microgels and their assembly have gained significant attention. Microgels are small enough to be injected through hypodermic needles, and micropores are formed by the interstitial space between microgels. When the microgels are mixed with the cells and crosslinked, the hydrogel provides a three dimensional environment that promotes cell spreading, migration, and cell–cell contact through the pore network^28^. Injectable microporous scaffolds have been previously used for cell delivery^29,30^, wound healing^31^, drug release, and 3D printing^32^, but their unique ability to naturally facilitate cell spreading and cell–cell contacts, and the resulting changes in stem cell differentiation have not been well studied.

**Figure 1:**
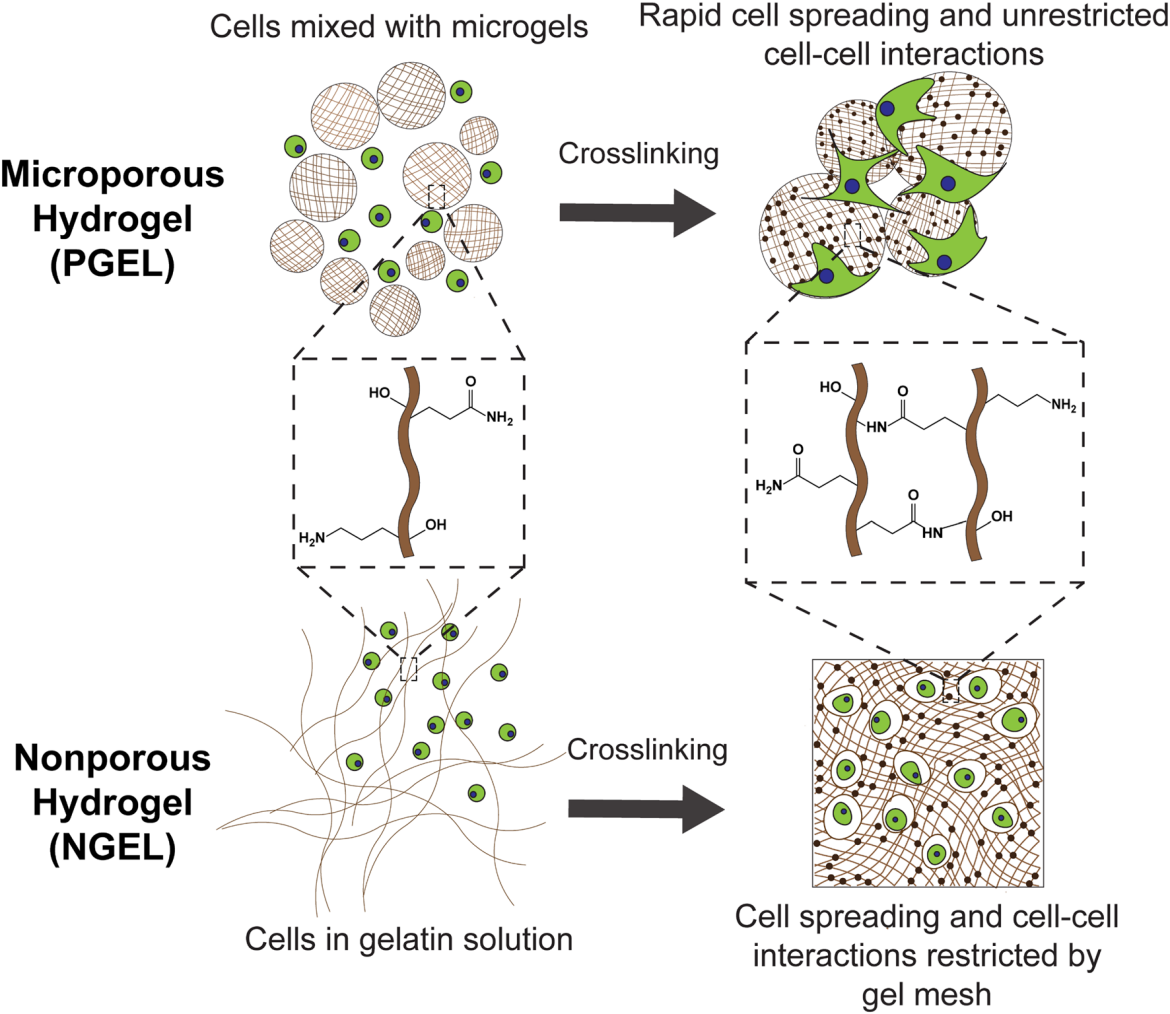
MSC encapsulation in the microporous (top) and nonporous (bottom) gelatin hydrogel. In the microporous hydrogel, MSCs are first mixed with physically crosslinked gelatin microgels, which situates the cells in the interstitial pore space. In the nonporous hydrogel, MSCs are dispersed in the gelatin solution. Both microporous and nonporous hydrogels are crosslinked by mTG which forms amide bonds between lysine and glutamine residues. Encapsulation in microporous hydrogels allows rapid cell spreading and cell–cell interactions regardless of scaffold stiffness. Cells encapsulated in stiff nonporous hydrogels are prevented from spreading by the surrounding dense polymer mesh. The differing 3D environment, while the material (i.e. gelatin) and mechanical stiffness are kept identical, results in different MSC proliferation and differentiation.

Previously we used a gelatin microgel-based platform as an injectable scaffold for wound healing, which improved migration of cells from excised cornea to the hydrogel interior in an ex-vivo study^33^. Microbial transglutaminase (mTG), which forms an amide bond between lysine and glutamine, was used to create crosslinks within and between microgels. The use of gelatin provides natural sites for cell adhesion and remodeling. Here we examine the use of this system to facilitate MSC osteogenic differentiation, and demonstrate that this system improves both MSC growth and osteogenic differentiation in comparison to a nonporous analog by promoting cell spreading and cell–cell interactions (Fig. 1). As mentioned earlier, the effects of micropores on MSC differentiation in the context of injectable hydrogels are poorly understood. These findings are further supported by genome-wide differences in gene expression investigated using RNA Sequencing (RNA-Seq).

## Results and discussion

### Hydrogel characterization

The gelatin microgels that are used in this research are polydisperse with the average diameter of ~ 200 µm^28^. Microgels are physically crosslinked and are stable in an aqueous solution at 25 °C for at least 24 h (Fig. S1). At 28 °C and 30 °C, microgels start to lose their structure after 30 and 5 min, respectively. Covalent crosslinking of the microgels by mTG results in a stable microporous hydrogel which remains stable throughout culture with MSCs at 37 °C for at least one month.

Void fraction, rheological properties, and equilibrium swelling ratio of swelled hydrogels were examined (Fig. S2). On average, swelled microporous hydrogels (**PGELs**) had a void fraction of 0.32 and had comparable stiffness as nonporous hydrogel (**NGEL**).The storage modulus remained stable over increasing angular frequency indicating stable chemical crosslinking^34^. On average, NGEL had a higher swelling ratio than PGEL, but this difference was not significant. In addition to these characterizations, detailed rheology of gelation, SEM images, injectability of PGELs and enzymatic degradation have been previously described^33^.

### Cell viability, proliferation and morphological changes

**Figure 2:**
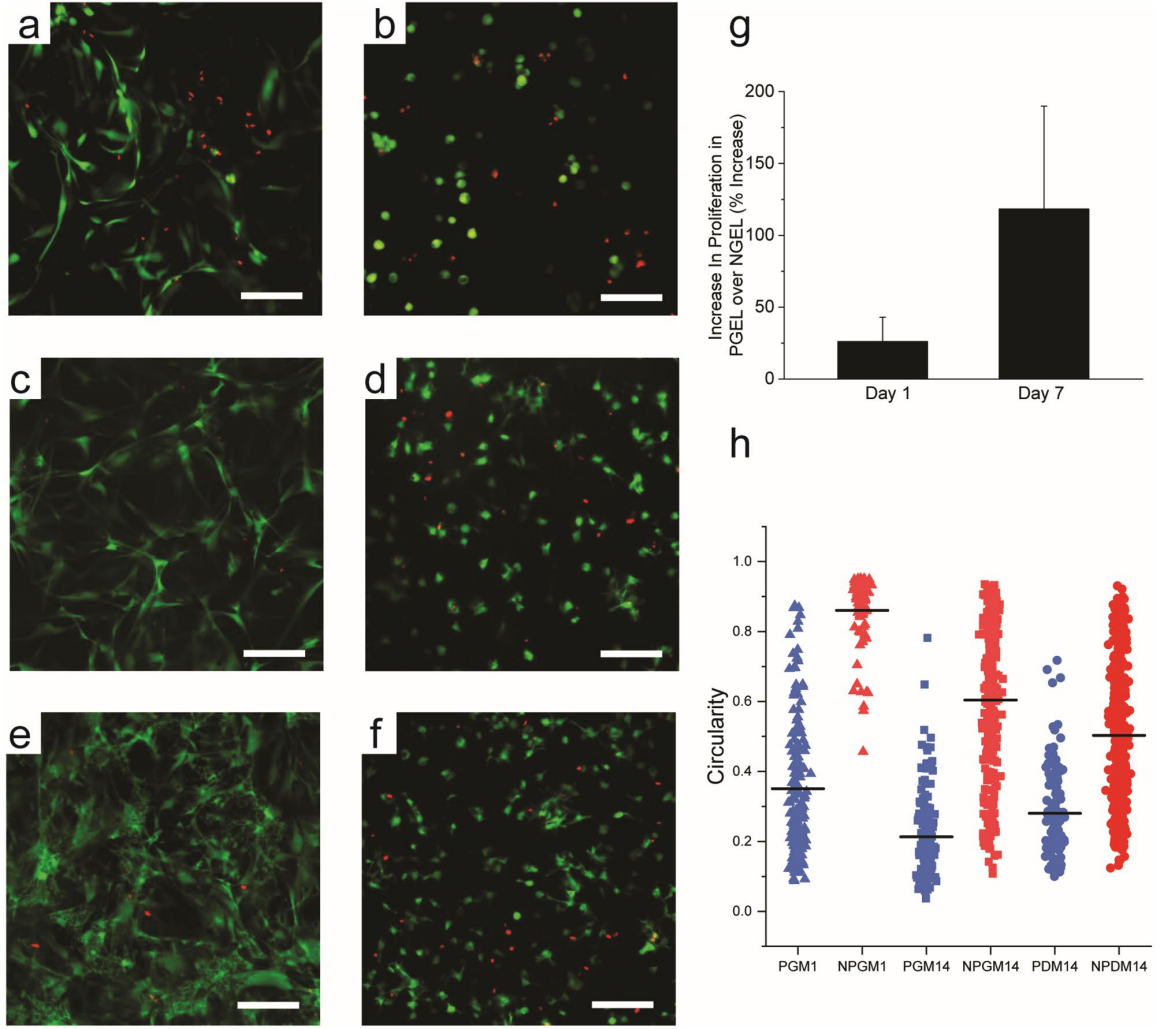
MSC growth in differing 3D culture conditions. Representative Z projections of living (green) and dead (red) cell staining of MSCs encapsulated in microporous (**a**, **c**, **e**) and nonporous (**b**, **d**, **f**) hydrogel in growth medium for day 1 (**a**, **b**), and day 14 (**c**, **d**), or incubated in osteogenic differentiation medium for 14 days (**e**, **f**). MSC proliferation was quantified by alamarBlue assay (**g**). Circularity of living cells in the microporous (P) and nonporous (NP) hydrogels incubated in growth medium (GM) for either 1 or 14 days, or differentiation medium (DM) for 14 days. *** p < 0.001 (Tukey’s HSD). Scale bar = 50 µm.

The potential for the PGELs to support cell encapsulation was explored through live/dead and alamarBlue proliferation assays, (Fig. 2) and Lactate Dehydrogenase (LDH) cytotoxicity assays (Fig. S3). Cells encapsulated in PGEL demonstrated high viability with robust cell spreading as early as 1 day post encapsulation (Fig. 2a), while cells in the NGEL remained highly spherical due to the entrapment by the surrounding polymers (Fig. 2b). The cells in the NGEL can fully spread only by matrix degradation or stress relaxation of the surrounding polymers. Live/dead assay on day 14 showed a continuation of these trends (Fig. 2c,d), and cells encapsulated in both conditions began to spread more compared with day 1. Cell proliferation in the PGEL was markedly higher than the NGEL (Fig. 2g). Cytotoxicity during the encapsulation process was low for both PGEL and NGEL (Fig. S3), demonstrating the biocompatibility of mTG crosslinking of gelatin. When the cells were cultured in osteogenic differentiation media, MSCs in the PGEL adopted a more complex random morphology (Fig. 2e) compared to the cells cultured in the growth media (Fig. 2c). In contrast, morphological changes of the cells encapsulated in the NGEL were less noticeable due to cell entrapment (Fig. 2d, Fig. 2f). Quantification of cell circularity confirms these observations. Notably, cell circularity in the PGEL was much lower than NGEL for all conditions due to cell spreading. Additionally, cells encapsulated in PGEL had increased circularity in response to differentiation, which is in contrast to the NGEL condition, where cell circularity decreased. MSC differentiation is affected by cell morphology^35,36^. Our findings highlight the importance of cell morphological changes during differentiation, and that when unobstructed, cells adopt morphology associated with these changes.

**Figure 3:**
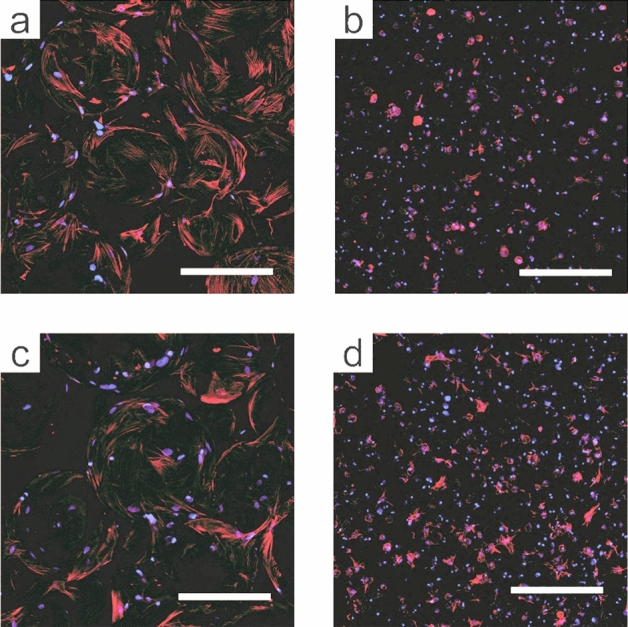
Cytoskeletal organization of encapsulated cells. Z-projection images of cell nuclei (blue) and actin cytoskeleton (red) after 1 (**a**, **b**) and 7 (**c**, **d**) days of culture, after encapsulation in the microporous (**a**, **c**) or nonporous (**b**, **d**) environment. Scale = 200 µm.

Morphology of encapsulated cells was further examined by staining for actin cytoskeleton and nuclei (Fig. 3). In accordance with Live/Dead imaging, encapsulated cells rapidly spread in the PGEL condition as early as 1 day after encapsulation (Fig. 3a). By comparison, cells encapsulated in NGEL displayed minimal spreading on day 1 (Fig. 3b). After 7 days of culture, minimal changes were observed in the spreading behavior in the PGEL condition (Fig. 3c), and cells had begun to spread in the NGEL condition (Fig. 3d). These results confirm the rapid adhesion and spreading behavior of cells encapsulated in PGEL, and rapid formation of actin stress fibers in this condition. The formation of actin stress fibers is known to enable mechanotransduction-mediated osteogenesis, suggesting PGEL may enhance MSC osteogenic differentiation^37^. In a prior study, in contrast with 2D systems, spreading of MSCs in nonporous hydrogels decreased with increasing stiffness^26^. The utility of PGEL circumvents this restriction by providing macroscopic, interconnected pore space for encapsulated cells.

### Osteogenic differentiation examined by EDS

Cell morphology, and calcium mineral deposition due to osteogenic differentiation, were observed under SEM and EDS (Fig. S4). Cells appear morphologically distinct, after incubation in osteogenic differentiation media. The increase in calcium and phosphorous in hydrogels incubated in osteogenic differentiation media is attributed to bone mineral deposition, which indicated encapsulated cells had successfully differentiated into osteoblasts.

### Biochemical characterization of osteogenic differentiation

**Figure 4:**
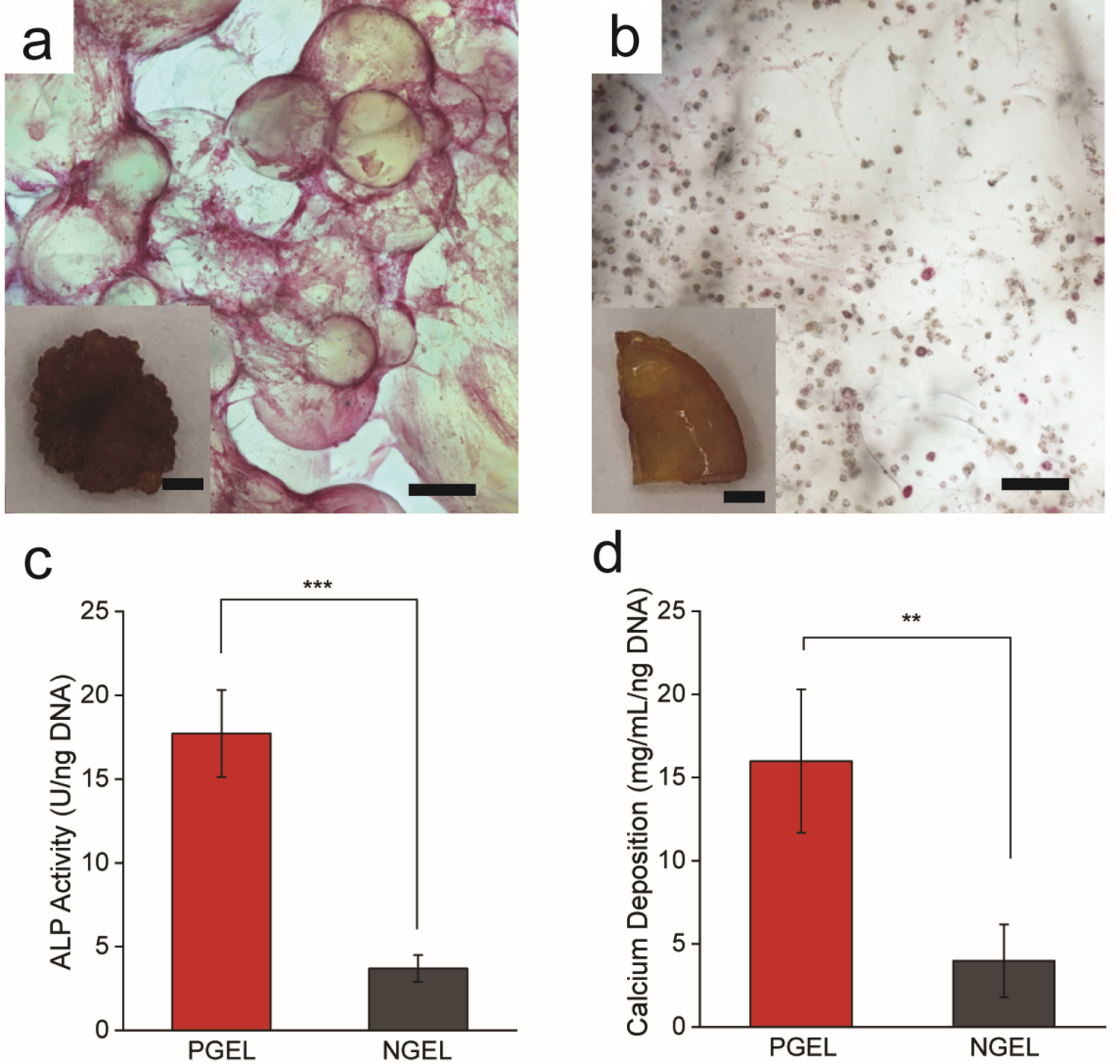
Microporous hydrogel enhances mesenchymal stem cell osteogenic differentiation. After 14 days of incubation in osteogenic differentiation medium: Alkaline phosphatase staining of cells encapsulated in (**a**) microporous (PGEL) and (**b**) nonporous (NGEL) hydrogels. (**c**) Alkaline phosphatase activity normalized to DNA content, and (**d**) calcium mineral deposition in equal volumes of cell-encapsulated hydrogels, normalized to DNA content. Scale bar = 200 µm, inset scale bar = 1 mm.

Osteogenic differentiation of MSCs encapsulated in PGEL and NGEL was examined by alkaline phosphatase (ALP) and calcium assays after 14 days of incubation in osteogenic differentiation medium (Fig. 4).

ALP staining shows a contrast between cells encapsulated in PGEL (Fig. 4a) and NGEL (Fig. 4b), where cells encapsulated in PGEL had significantly higher ALP activity. ALP is an enzyme involved with the mineralization of bone tissue, and is a marker of early MSC osteogenic differentiation^38^. This microscopic observation is consistent with quantitative results, which show ALP activity and calcium deposition increased by about a factor of 4 on a per cell basis (Fig. 4c, d) for cells encapsulated in PGEL in comparison to NGEL. Calcium deposition is indicative of mature osteoblasts, demonstrating that encapsulation in PGEL improved mineral deposition over the culture period.

Considering the identical material and comparable stiffness of PGEL and NGEL^33^, these results highlight the importance of differing 3D micro-environments for the control of MSC osteogenesis. More specifically, PGELs allow rapid morphological changes of the encapsulated cells and direct cell–cell physical contacts through the interconnected micropore network, which may have promoted osteogenesis and calcium mineral deposition. Whether the differing pore structure affects nutrient transport to encapsulated cells is unclear, as cells encapsulated in PGEL are clustered at a high local cell density in the pore space, compared with homogeneous cell distribution in NGEL.

### Transcriptomic analysis by RNA-seq

**Figure 5:**
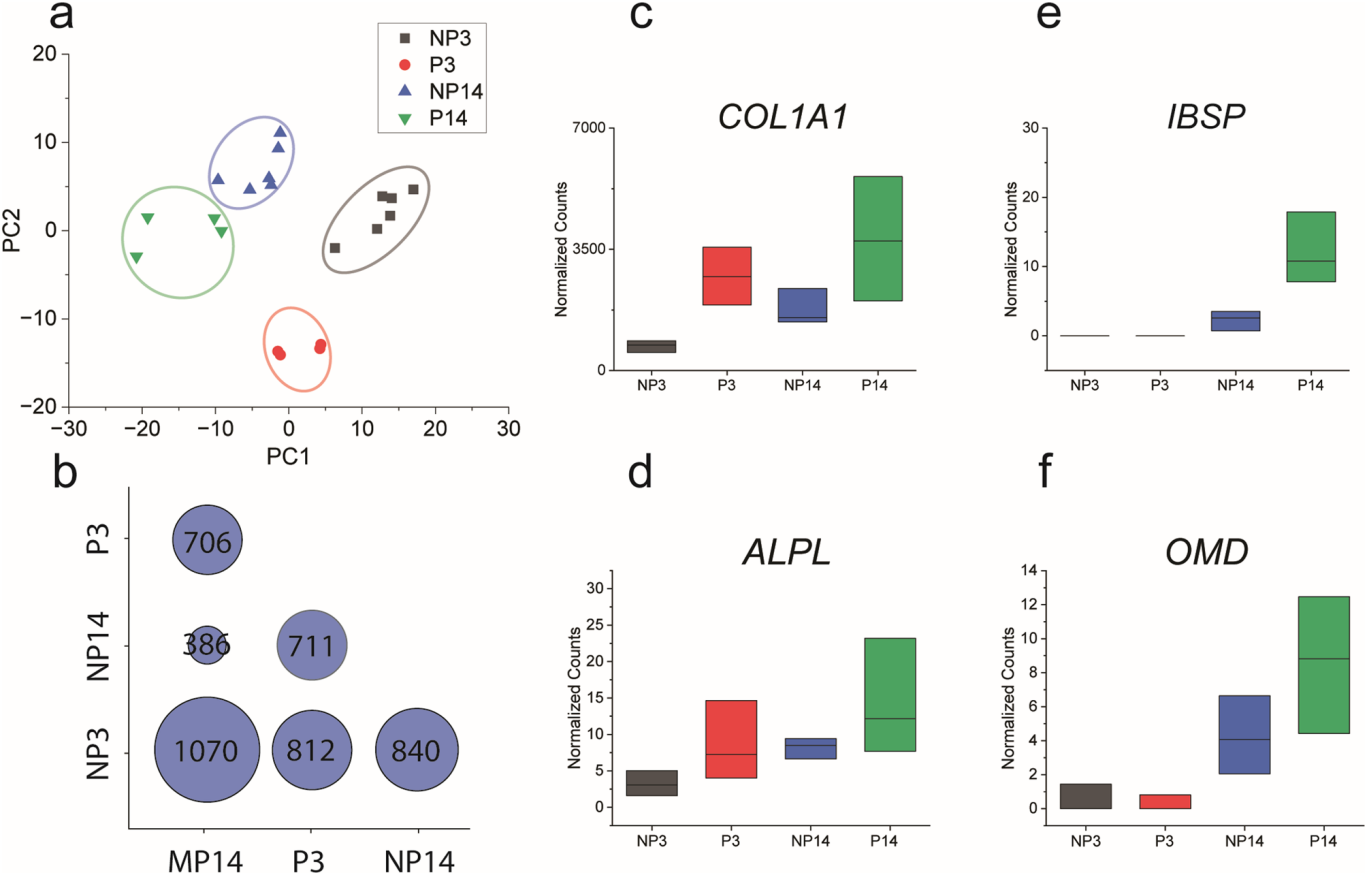
Differential gene expression identified by RNA-Seq. (**a**) PCA of sample set. PC1 and PC2 account for 43% and 22% of variance, respectively. (**b**) Number of differentially expressed genes between groups, comparing culture condition and time. (c-e) Expression of genes directly related to osteogenesis, collagen type I alpha chain 1 (*COL1A1*), integrin binding sialoprotein (*IBSP*), alkaline phosphatase, biomineralization related (*ALPL*), and osteomodulin (*OMD*). Abbreviations: NP3 (Nonporous, day 3), P3 (Microporous, day 3), NP14 (Nonporous, day 14), P14 (Porous, day 14). Data shown are median, bounded by the interquartile range.

**Figure 6:**
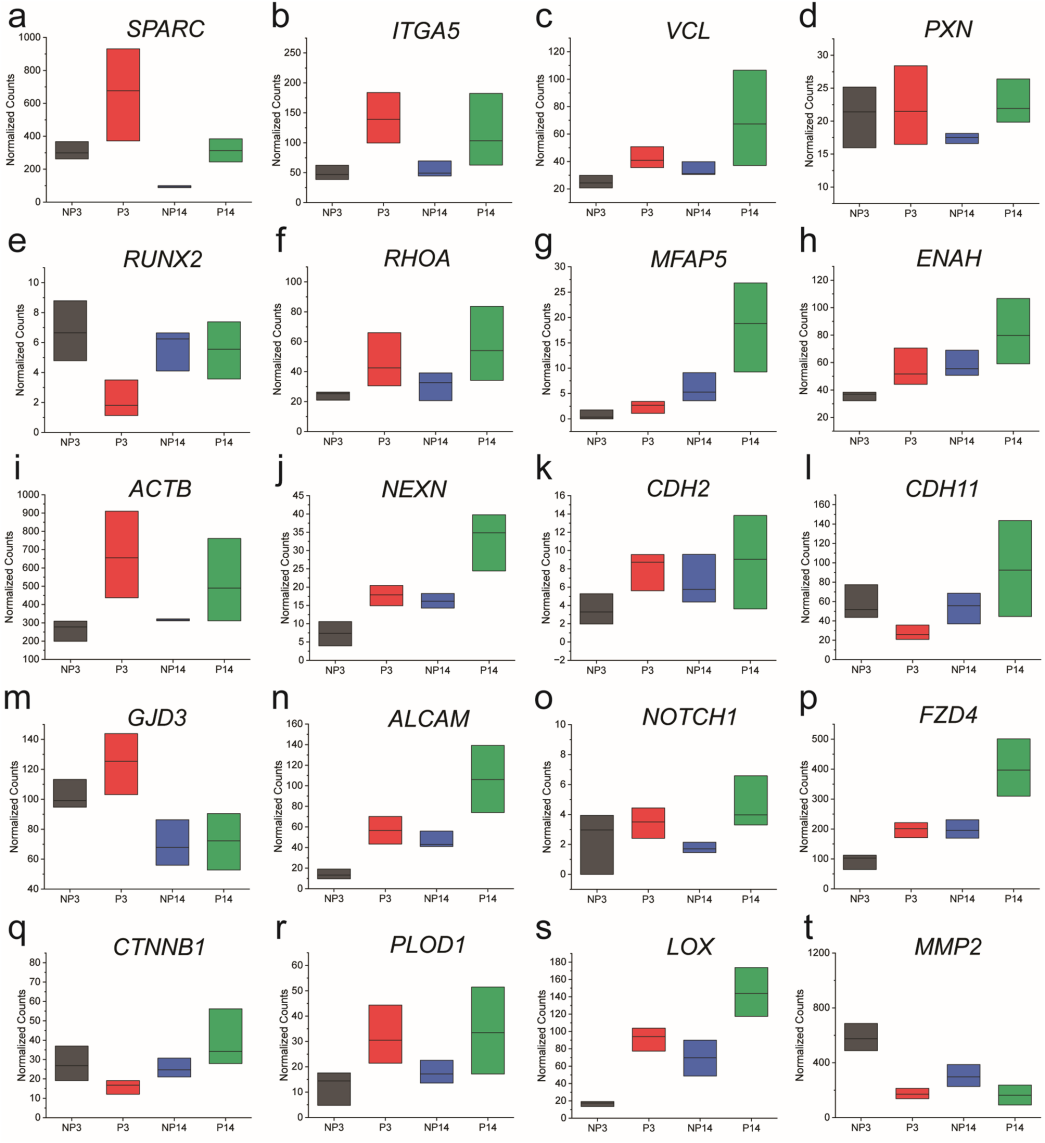
Gene expression for genes of interest in different 3D environments and time points, for genes related to osteogenesis (**a**), mechanotransduction (**b**, **c**, **d**, **e**, **f**), cystoskeleton production and organization (**g**, **h**, **I**, **j**), cell–cell connections (**k**, **l**, **m**, **n**, **o**, **p**, **q**), extracellular matrix production and matrix remodeling (r, s, t). Data is separated between culture condition and time point (NP3 = nonporous day 3, P3 = porous day 3, NP14 = nonporous day 14, P14 = porous day 14). (**a**) Secreted Protein Acidic and Cysteine Rich (SPARC), (**b**) Integrin Subunit Alpha 5 (ITGA5), (**c**) Vinculin (VCL), (**d**) Paxillin (PXN), (**e**) Runt-related Transcription Factor 2 (RUNX2), (**f**) Ras Homolog Family Member A (RHOA), (**g**) Microfibril Associated Protein 5 (MFAP5), (**h**) ENAH Actin Regulator (ENAH), (**i**) Actin Beta (ACTB), (**j**) Nexilin F-Actin Binding Protein (NEXN), (**k**) Cadherin 2 (CDH2), (**l**) Cadherin 11 (CDH11), (**m**) Gap Junction Protein Delta 3 (GJD3), (**n**) Activated Leukocyte Cell Adhesion Molecule (ALCAM), (**o**) Notch Receptor 1 (NOTCH1), (**p**) Frizzled Class Receptor 4 (FZD4), (**q**) catenin beta 1 (CTNNB1), (**r**) Procollagen-Lysine, 2-Oxoglutarate 5-Dioxygenase 1 (PLOD1), (**s**) Lysyl Oxidase (LOX), (**t**) Matrix Metalloproteinase 2 (MMP2).

RNA-Seq was used to examine the changes in gene expression of MSCs encapsulated in the different 3D environments. RNA from MSCs encapsulated in PGEL and NGEL was extracted at 3 days (P3, NP3), and 14 days (P14, NP14) after encapsulation to assess early and late differentiation (Figs. 5,6). PCA analysis (Fig. 5a) shows a clear trend based on sample condition and time, indicating that gene expression changed substantially depending on the 3D environment, and on the duration of differentiation. The number of differentially expressed genes between groups (Fig. 5b) aligns with PCA analysis, confirming the central role the 3D environment played in differential gene expression. Genes commonly related to osteogenic differentiation (Fig. 5c-f) show a trend that osteogenic differentiation was increased in the PGEL, and increased over incubation time. Integrin binding sialoprotein (*IBSP*) expression (Fig. 5e) is notable as a late stage marker of osteogenesis. These results confirm the increase in osteogenesis for cells encapsulated in the PGEL condition. Expression data for selected genes related to osteogenesis, cell adhesions, cytoskeletal organization, cell–cell connections, ECM remodeling and deposition are shown in Fig. 6.

#### Osteogenesis genes

Osteonectin (SPARC) is a protein involved in calcium mineral deposition, which had increased expression in PGEL, providing further evidence of the increase in osteogenesis for these cells.

#### Cell adhesion, focal adhesion genes

Increase in Integrin Subunit Alpha 5 (*ITGA5*) expression, related to integrin α_5_β_1_ (one of the primary integrins involved in binding to gelatin), in PGEL is consistent with an increase in cell spreading as visualized in confocal images^39^. However, expression of proteins related to focal adhesions and focal adhesion-mediated signaling overall did not show a clear trend (*PXN*, *RUNX2*, *YAP1*), though vinculin (*VCL*) expression was upregulated in the PGELs, and with increasing culture length. In similar 3D matrices, it was previously reported that differences in gene expression of mechanotransduction-related genes was diminished as length of culture increased^40^, which could explain this trend. Additionally, while cell spreading is higher for cells in PGEL, substrate stiffness is similar between PGEL and NGEL, which may have resulted in insignificant differences in the expression of these genes, due to the well-known relationship between substrate stiffness and focal adhesion formation^41^.

#### Cytoskeletal organization genes

Gene expression related to cytoskeletal organization (*MFAP5*, *ENAH*, *ACTB*, *NEXN*) shows a general trend of increased expression for cells encapsulated in PGEL, and an increase in expression over the culture period, likely as a product of increased cell spreading.

#### Cell–cell connection genes

Among the genes related to cell connections, an increase in *CDH11* expression in PGEL was noted. On 2D surfaces, higher *CDH11* expression correlated with higher osteogenesis of MSCs^19^. *CDH11* expression was constant for cells encapsulated in the NGEL, though expression at day 3 was higher than in PGEL. Among gap-junction proteins, which have been previously implicated to regulate MSC differentiation^42^, *GJD3* was highly expressed, and had increased expression for cells in PGEL.

#### Wnt/Notch signaling genes

*CTNNB1*, *NOTCH1*, *FZD4* are involved in cell signaling pathways (Wnt/Notch). We hypothesize that over the culture period, cells encapsulated in PGEL increased the number of cell–cell connections as cell density in the hydrogel increased, leading to increased expression of cell–cell connection-related genes, and potentially associated pathways, such as the Notch pathway. However, gene expression for cells encapsulated in the NGEL were generally prevented from making these connections, and expression of cell–cell connection related genes remained constant as a result. Additionally, *CTNNB1* and *FZD4* participate in Wnt signaling, which mediates mechanical stretching-induced osteogenesis^43^, which may have been influenced by the differing microenvironments, and has been previously implicated to mediate osteogenesis for MSC aggregates on differing biomaterial substrates^44^.

#### ECM remodeling genes

Lysyl hydroxylase 1 (*PLOD1*) and lysyl oxidase (*LOX*) are involved with collagen production, indicating ECM deposition was increased in PGEL in comparison to NGEL. We hypothesize that the open pore space may enable more rapid production of ECM, as cells in this condition do not need to degrade the surrounding matrix. Matrix metalloproteinase 2 and 9 (MMP2 and MMP9) are gelatinases, some of the primary means for cells to degrade gelatin. Cells encapsulated in NGEL may need to degrade the surrounding polymer mesh for division, spreading, and new ECM production, likely leading to the observed increased production of MMP2. MMP9 expression was not detected in any sample groups by RNA-seq. In addition to the selected genes, other genes of interest are shown in Fig S5. Statistical significance of all graphed gene expression comparisons are displayed in Fig S6.

**Figure 7:**
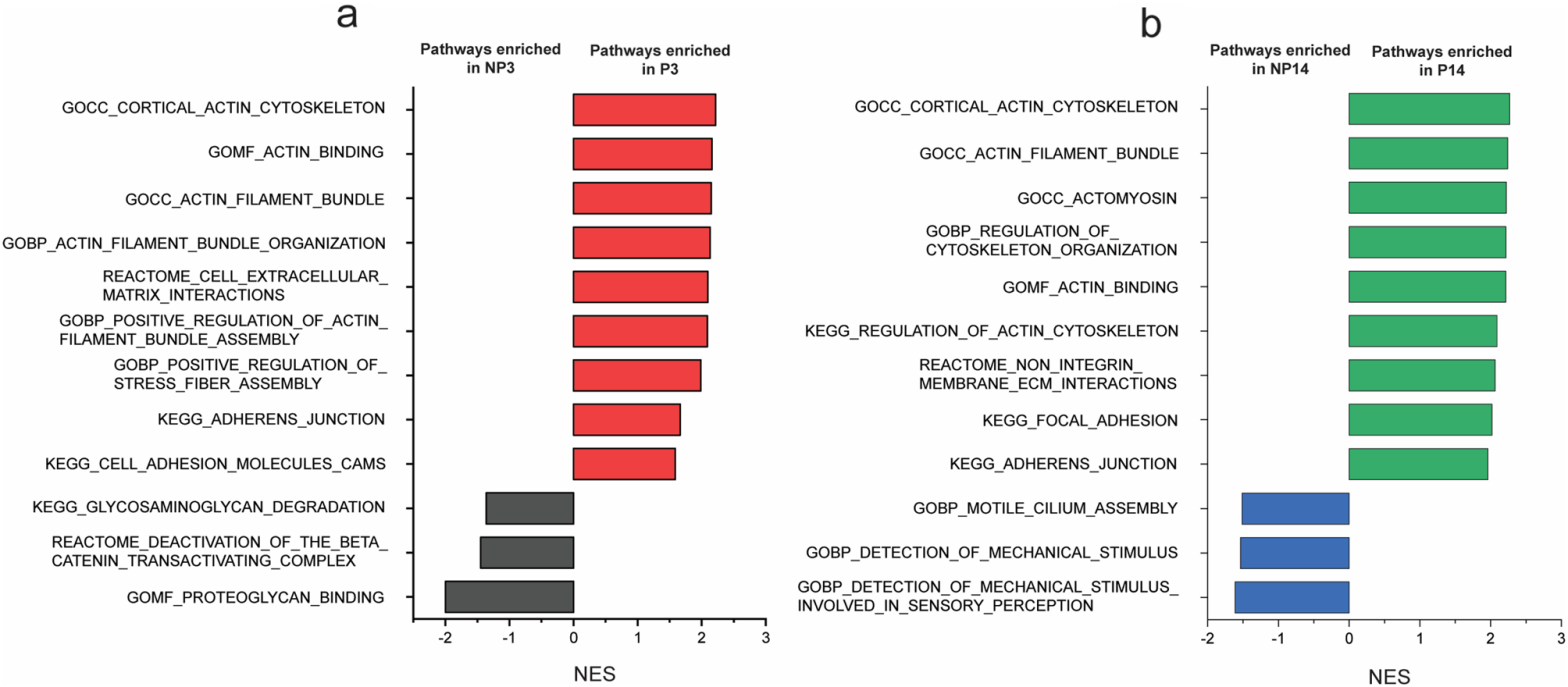
Normalized Enrichment Score (NES) of selected upregulated pathways in pairwise comparisons between (**a**) nonporous day 3 (NP3) and porous day 3 (P3), and (**b**) nonporous day 14 (NP14) and porous day 14 (P14), given by GSEA.

GSEA was used to investigate differences in pathway activity between sample groups. The most recently updated KEGG, REACTOME, and GO databases were used. For each pairwise comparison, top pathways sorted by normalized enrichment score (NES) were plotted (Fig S7), and selected pathways relevant to our investigation are shown in Fig. 7. In addition, the full data table is supplied as additional supplementary information. While several highlighted pathways appear to be unrelated to MSC differentiation, many pathways relevant to cell behavior scored highly, in agreement with our observations at the single gene level. On day 3, pathways related to integrin-cell surface interactions, actin and laminin binding, focal adhesions, and adherens junctions have higher gene expression in the PGEL condition than in the NGEL condition (Fig. 7a). On day 14, many identified pathways continue to be upregulated in comparison to the NGEL condition, including adherens junctions, focal adhesions, actin assembly, and integrin-cell surface interactions, indicating the effect of the 3D environment on encapsulated cells continued to affect cell behavior throughout the culture period (Fig. 7b). In the NGEL condition on day 3, pathways related to ECM degradation and binding (Fig. 7a), and on day 14, mechanosensing (Fig. 7b), were highlighted.

## Conclusions

The inverse relationship between hydrogel stiffness and cell spreading in injectable hydrogel culture prompts the investigation of injectable hydrogels that can better direct MSC differentiation. In this work we examined the use of a microporous injectable hydrogel to promote MSC osteogenic differentiation in comparison to a conventional nonporous injectable hydrogel. PGEL promoted cell spreading, and cell–cell connections of the encapsulated MSCs because of the interconnected pore network, and induced more efficient osteogenic differentiation than NGEL. RNA-seq identified genes and gene pathways differentially expressed as a result of encapsulation in differing microenvironments. Additionally, this work demonstrates a functional injectable system which can provide a stiff 3D environment to MSCs, yet facilitates cell spreading, elongation, and cell–cell connections, in contrast with contemporary injectable systems. This research demonstrates the potential use of microgel-based injectable hydrogel systems for bone repair, and points to the possibility of bone tissue regeneration in vivo considering that other MSC delivery systems resulted in significant bone tissue regeneration in various animal models^45–47^.

## Materials and methods

### Materials

Bone marrow human mesenchymal stem cells (MSCs) were purchased from ATCC. Minimum Essential Medium α (MEM-α), fetal bovine serum (FBS), penicillin/streptomycin (pen/strep), LDH assay, AlamarBlue assay, actin red 555 and 4',6-Diamidino-2-Phenylindole Dihydrochloride (DAPI), Quant-iT dsDNA assay, Point Scientific Calcium Assay and Live/Dead assay were purchased from Thermo Fisher Scientific. 300 Bloom gelatin type A was obtained from Sigma Aldrich. Osteogenic differentiation medium was purchased from Promocell. Alkaline phosphatase (ALP) staining kit, and ALP assay were obtained from Abcam. Activa Ti microbial transglutaminase was obtained from Ajinomoto.

### Microgel production

Gelatin microgel production has been previously reported^33^. In brief, 20 mL 10% w/v gelatin in DI H_2_O was added to 200 mL olive oil at 55 °C and stirred for 1 h. To create physically crosslinked microgels, the temperature was dropped through addition to an ice bath for 30 min while stirring. 100 mL precooled acetone was added to dehydrate the microgels and aid in filtration, mixing for 30 min. Microgels were separated by vacuum filtration, washed with additional acetone, then sterilized in 70% ethanol, and freeze dried, before being used in cell experiments. The produced gelatin microgels have an average diameter of 253 µm in diameter after swelling^33^.

### Hydrogel characterization

Gelatin microgels were incubated in PBS at 25, 28, and 30 °C to evaluate their stability. Microscope images were taken periodically, at 1, 5, and 30 min, and after 24 h incubation to assess retention of microgel structure.

For experiments with swelled hydrogels, 100 mg of microgel was mixed with 1 mL PBS in a 12 well plate well, for rehydration. Then, 250 µL 20% w/v mTG solution was mixed with the swelled microgels to a final concentration of 8% gelatin and 4% mTG. The hydrogels were incubated at 37 °C for 1 h for crosslinking. For nonporous hydrogels, 1 mL of 10% gelatin solution in PBS was mixed with 250 µL mTG solution, before incubation at 37 °C for 1 h. After crosslinking, hydrogels were submerged in excess PBS for 24 h before experiments. Void fraction measurement was performed by imaging the background autofluorescence which differentiates between the microgels and pore space, then performing object counting analysis of 2D slices of hydrogel. Rheological properties of swelled hydrogels were determined by angular frequency sweep at 37 °C, between 1 and 50 rad/s, at an oscillatory stress of 2 Pa. The mass of swollen hydrogels was compared with mass after lyophilization to obtain equilibrium swelling ratio for PGEL and NGEL.

### Cell encapsulation

MSCs were cultured on T-75 flasks prior to cell encapsulation in MSC growth medium (MEM-α, 10% FBS, 1% pen/strep). For cell encapsulation experiments, 20 mg of microgel was mixed with 150 µL MEM-α in a 48 well plate well, for rehydration. Then, 50 µL of cell suspension and 50 µL filter-sterilized mTG solution was mixed with the swelled microgels to a final concentration of 8% gelatin, 1 × 10^6^ cells/mL, and 4% mTG. The hydrogels were incubated at 37 °C for 1 h for crosslinking. For nonporous hydrogels, 150 µL of 13.3% gelatin solution in MEM-α was mixed with 50 µL of cell suspension and 50 µL mTG solution, before incubation at 37 °C for 1 h. After crosslinking, cell-encapsulated hydrogels were moved to 24 well plates, and fed daily with 1.5 mL of media. For cell growth experiments, MSC growth medium was used, and for differentiation experiments, osteogenic differentiation medium was used after 24 h of incubation in growth medium. Cells of passage 3 were used for all experiments.

### Proliferation/cytotoxicity experiments

Cell-encapsulated hydrogels were tested for LDH activity 24 h after encapsulation to assess the cytotoxicity as a result of the encapsulation process. Cells seed on TCPS were used as the negative control, and for the positive control after treatment with lysis buffer. Cell proliferation was measured using alamarBlue assay at 24 h and 7 days after encapsulation, where cells seeded on TCPS were used as a positive control.

### Live/dead assay

Live/Dead assay was performed on cell-encapsulated hydrogels at 1, 7, and 14 days after encapsulation, for hydrogels incubated with either growth medium or osteogenic differentiation medium to monitor cell growth and morphological changes as a result of differentiation. Hydrogels were incubated with HBSS containing calcein-AM and ethidium homodimer to visualize living and dead cells respectively, for 1 h before imaging (Nikon A1R HD). 3D sections of cell encapsulated hydrogels were imaged, and images were processed and converted to Z projections using ImageJ. Cell circularity was calculated using the ImageJ software.

### Actin cytoskeleton imaging

Cells encapsulated in porous and nonporous gels were fixed in 4% paraformaldehyde for 1 h after 3 and 14 days of culture in growth medium. Cells were permeabilized in 1% Triton X-100 for 1 h, stained with DAPI and actin red 555 overnight at 4 °C. Constructs were washed in PBS for 1 h before confocal imaging. Images were analyzed using ImageJ.

### SEM/EDS

Cell-encapsulated hydrogels were fixed with 2.5% glutaraldehyde solution in PBS, then moved to ethanol by serial dilution, and critical point dried. Hydrogels were mounted and sputter coated with Au/Pd before SEM/EDS.

### Alkaline phosphatase staining and quantitative assay

Alkaline phosphatase staining kit (Abcam) was used according to the manufacturer protocol. Cell encapsulated constructs after 14 days of incubation in osteogenic differentiation medium were fixed with the provided fixative, then stained for 30 min, before washing 4 × with PBS for 1 h to remove the discoloration of the hydrogel. High magnification images were taken using the DS-Ri2 camera attachment for the confocal microscope.

ALP assay (Abcam) was used to determine ALP activity in cell-encapsulated constructs after 14 days of incubation in osteogenic differentiation medium, normalized to measured dsDNA content. Cell-encapsulated constructs were mechanically disrupted using a tissue homogenizer, and incubated in RIPA lysis buffer. Samples were centrifuged, and lysis buffer was removed for ALP and dsDNA concentration measurements, which were calculated from standard curves.

### Calcium assay

Cell encapsulated hydrogels were homogenized using a rotor homogenizer, and after removal of cellular components for dsDNA/ALP assay measurement, 50 µL 12 N HCl was added to cell encapsulated constructs for 72 h at 4 °C to dissolve deposited calcium. A Pointe Scientific Calcium assay was used to measure calcium concentration. Samples were diluted in PBS to fall in the linear range of the assay before measuring. As pH was observed to influence the assay results, all samples were diluted until sample pH was neutral.

### RNA sequencing

RNA extraction was carried out using the RNeasy Plus Mini kit from Qiagen according to the manufacturer’s protocol. Cell encapsulated constructs were homogenized with a rotor homogenizer before extracting the RNA. Due to the lower RNA yield for nonporous samples, a higher sample number was used. Isolated RNA was frozen at –80 °C until use. Extracted RNA was supplied to the UNH Hubbard Center for Genome Studies for mRNA isolation, library preparation, and sequencing. Sequencing was performed on Illumina HiSeq2500. Paired end reads were trimmed using trimmomatic^48^, aligned to the human genome using STAR^49^, and raw reads were counted using HT-seq^50^. Data normalization and analysis was done in R using the Deseq2 package. PCA was performed using a regularized log dataset using tools from the Deseq2 package^51^. Gene set enrichment analysis (GSEA) was performed using the software provided by the Broad Institute^52,53^. Pathway analysis for pairwise comparisons were done using the REACTOME^54^, GO^55,56^, and KEGG^57,58^ pathway databases.

### Statistical analysis

Quantitative data other than RNA-Seq data are represented as means, and error bars represent standard deviations (for image analysis data, n = 3. For proliferation/cytotoxicity, ALP and calcium assay, n = 4. For RNAseq, n = 4 for porous samples, and 6 for nonporous samples). For comparisons with only two groups, a student’s t-test was used to determine statistical significance, where p < 0.05 was considered statistically significant (* p < 0.05, ** p < 0.01, *** p < 0.001. For multiple comparisons, Tukey’s HSD was used. RNA-seq data is presented with median (line) and the interquartile range due to the non-normal distribution of the data.

### Supplementary Information

Supplementary Information.

## Data Availability

The RNA-seq datasets generated and analyzed during the current study are available in the Gene Expression Omnibus (GEO) repository, GSE263939**.** Additional data analyzed in this study is available from the corresponding author, upon request.
