# Supplementary material for: Enhanced osteogenesis of mesenchymal stem cells encapsulated in injectable microporous hydrogel
Source: Sci Rep. 2024 Jun 25;14:14665. doi: 10.1038/s41598-024-65731-9 (PMC11199573; doi:10.1038/s41598-024-65731-9)
Supplement: Supplementary file 1 — Supplementary Information. [file 41598_2024_65731_MOESM1_ESM.pdf]

Supporting Information for:

Enhanced Osteogenesis of Mesenchymal Stem Cells Encapsulated in Injectable Microporous Hydrogel

*Seth D. Edwards, Mrinal Ganash, Ziqiang Guan, Jeil Lee, Young Jo Kim, Kyung Jae Jeong**

Department of Chemical Engineering, University of New Hampshire, Durham, NH, 03824, United States

* Corresponding author contact: KyungJae.Jeong@unh.edu

This document includes: Supplementary figures S1 – S7 (pages 1 – 17)

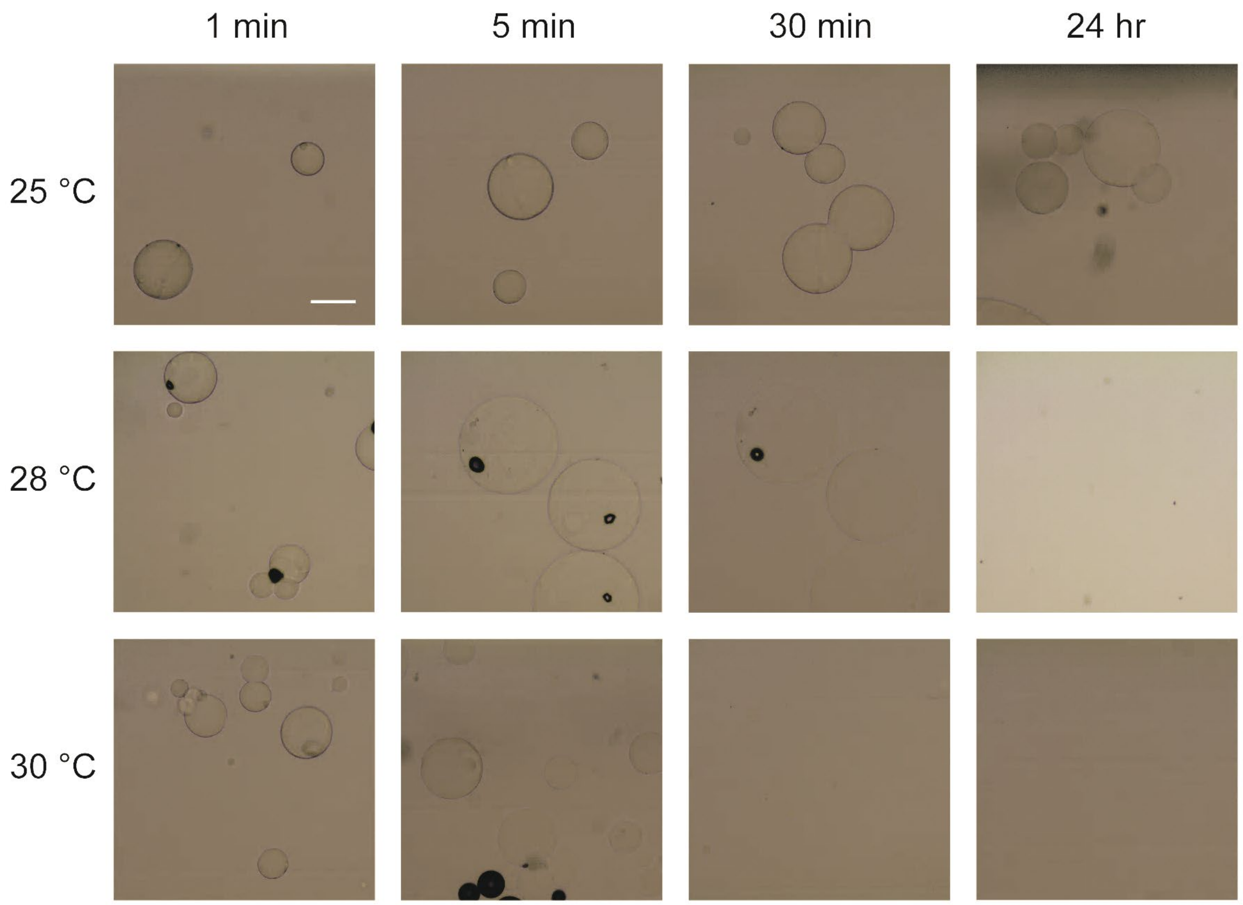

Figure S1. Stability of gelatin microgels in PBS. Brightfield microscope images of microgels after incubation in PBS at 25, 28, and 30 °C after 1, 5, and 30 minutes, or 24 hours incubation time. Scale = 200 μm.

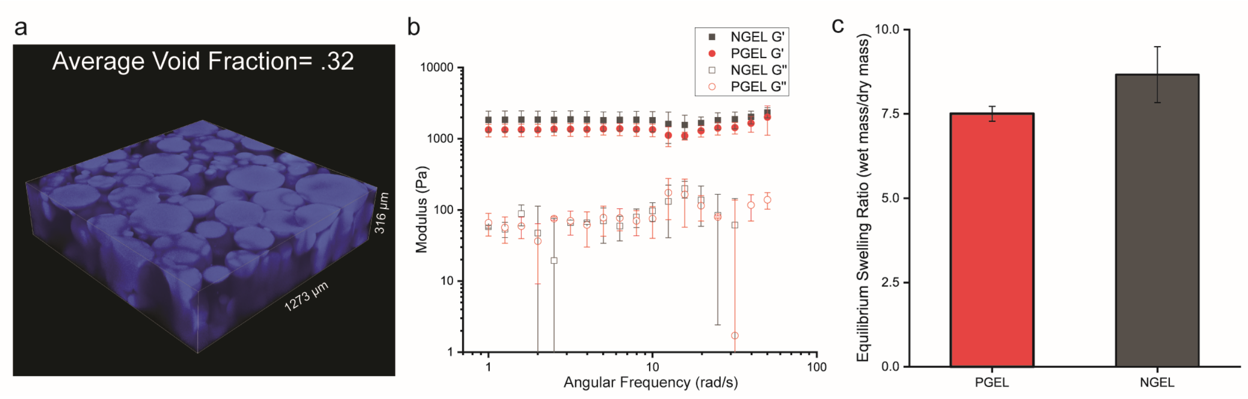

Figure S2. Characterization of swelled gelatin microporous hydrogel. a) 3D visualization of microporous hydrogel (PGEL) porosity, b) frequency sweep rheology of PGEL and nonporous gelatin hydrogel (NGEL), showing average and standard deviation for storage modulus (G') and loss modulus (G''), and c) equilibrium swelling ratio of PGEL and NGEL.

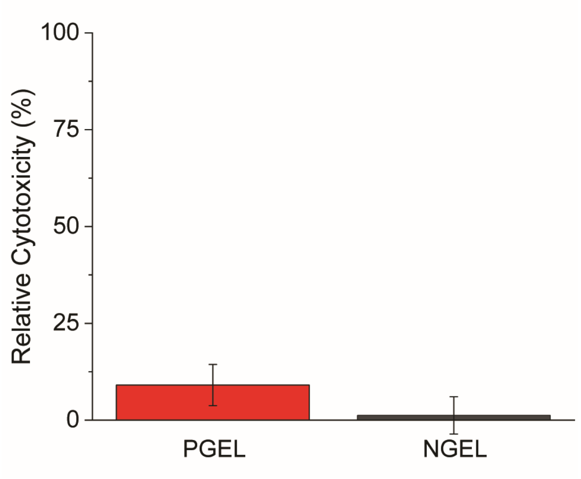

Figure S3. Cytotoxicity of the encapsulation procedure. LDH assay of cells 24 hours after encapsulation in either the porous (PGEL), or nonporous (NGEL) condition.

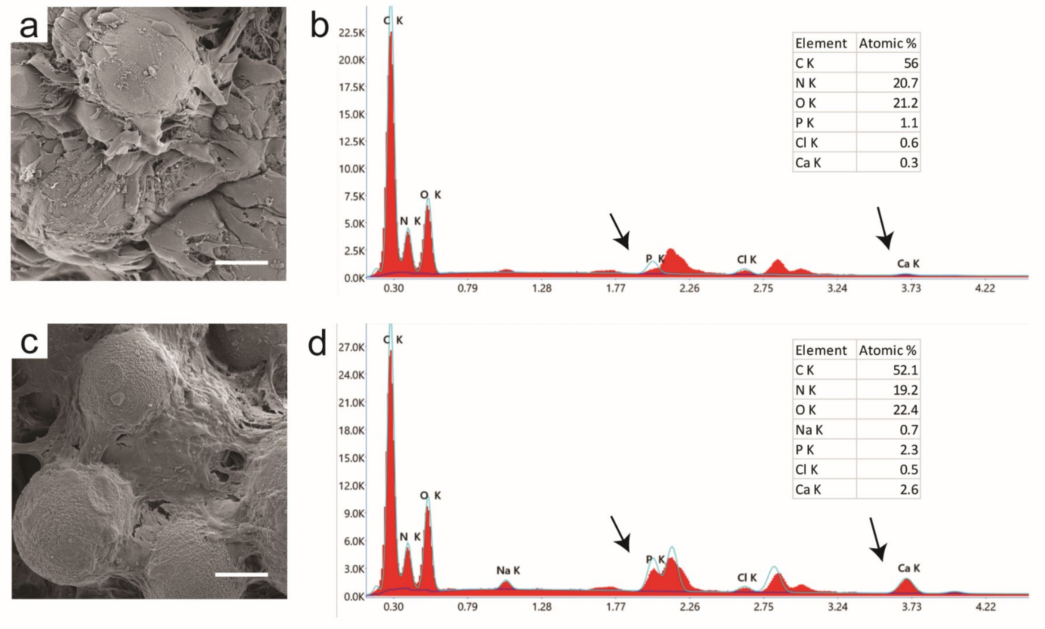

Figure S4. Cell morphology in microporous hydrogels and initial evidence of mineral deposition. SEM and EDS chemical composition report for cells grown for 14 days in (a, b) growth medium or (c, d) osteogenic differentiation medium. Arrows denote peaks of interest for mineralization, and atomic weight percentage is inset. Scale = 50 μm.

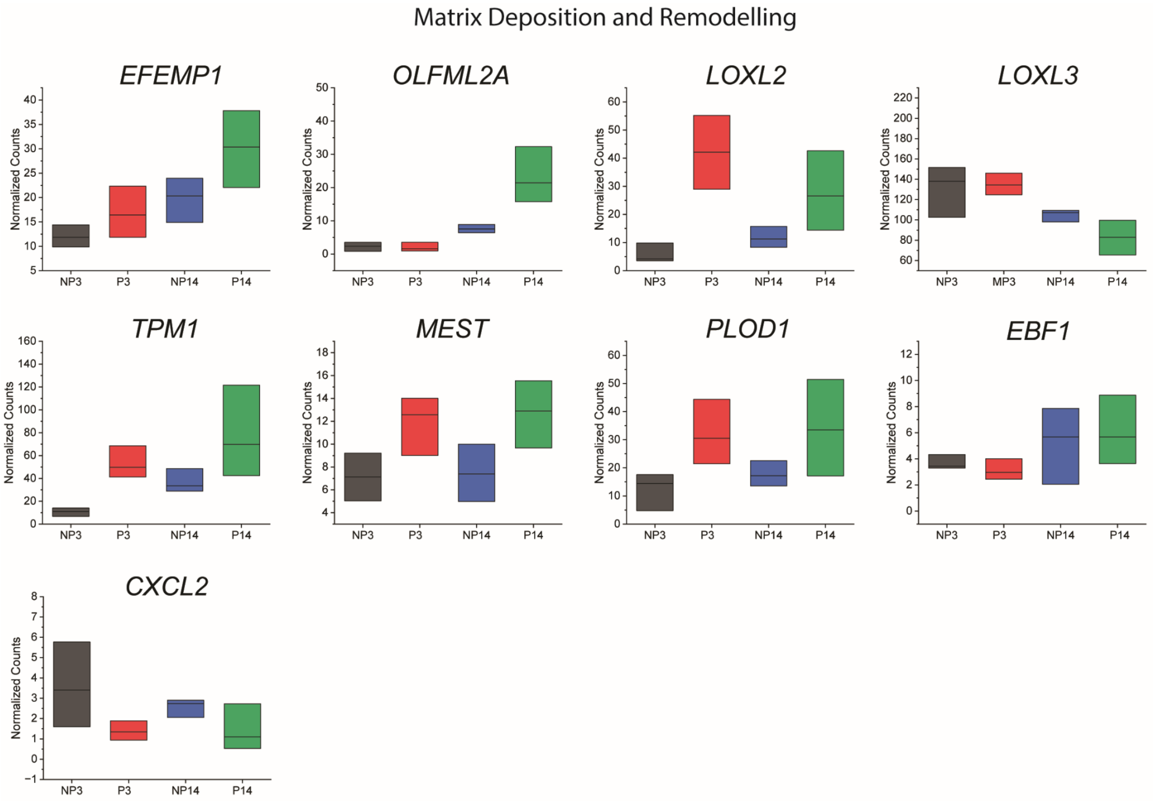
Matrix Deposition and Remodelling
EFEMP1
OLFML2A
LOXL2
LOXL3
TPM1
MEST
PLOD1
EBF1
CXCL2
Normalized Counts
NP3
P3
MP3
NP14
P14

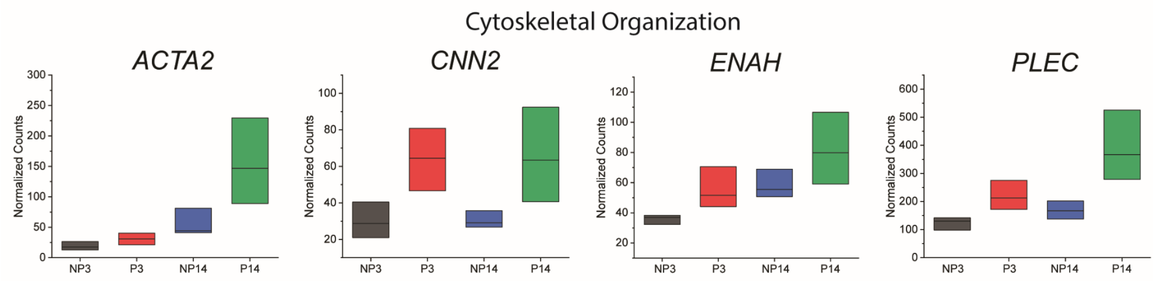
Cytoskeletal Organization
ACTA2
CNN2
ENAH
PLEC
Normalized Counts
NP3
P3
NP14
P14

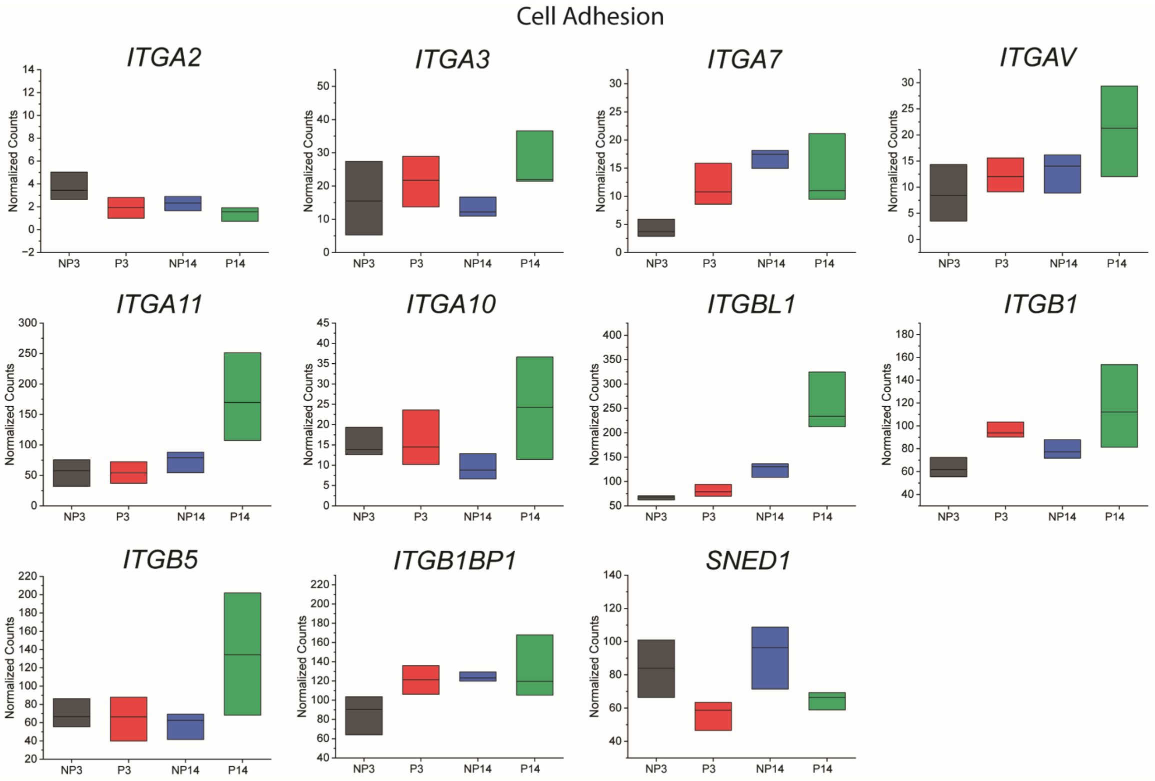

Cell Adhesion
ITGA2
ITGA3
ITGA7
ITGAV
ITGA11
ITGA10
ITGBL1
ITGB1
ITGB5
ITGB1BP1
SNED1
Normalized Counts
NP3
P3
NP14
P14

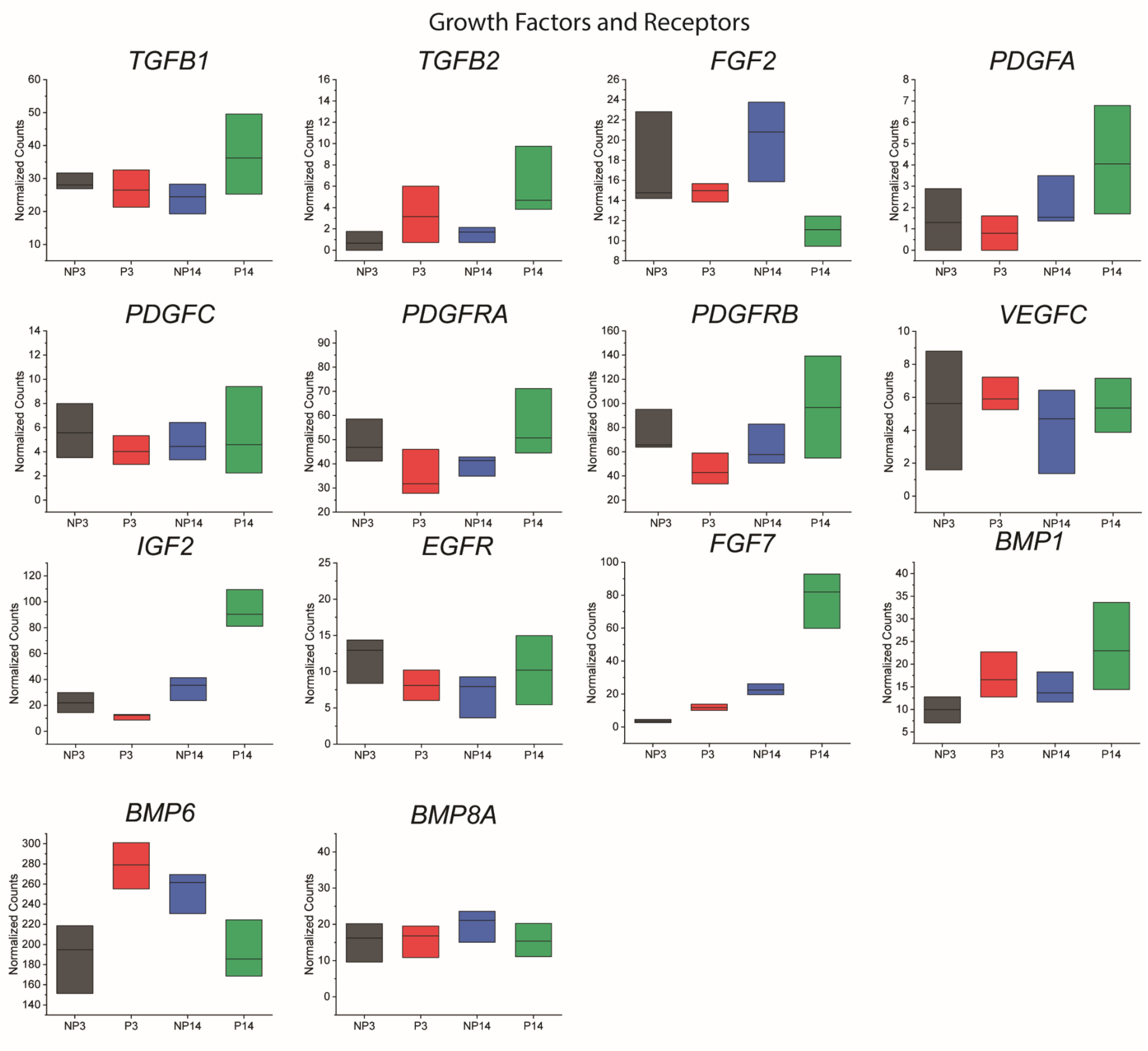
Growth Factors and Receptors
TGFB1
TGFB2
FGF2
PDGFA
PDGFC
PDGFRA
PDGFRB
VEGFC
IGF2
EGFR
FGF7
BMP1
BMP6
BMP8A
Normalized Counts
NP3
P3
NP14
P14

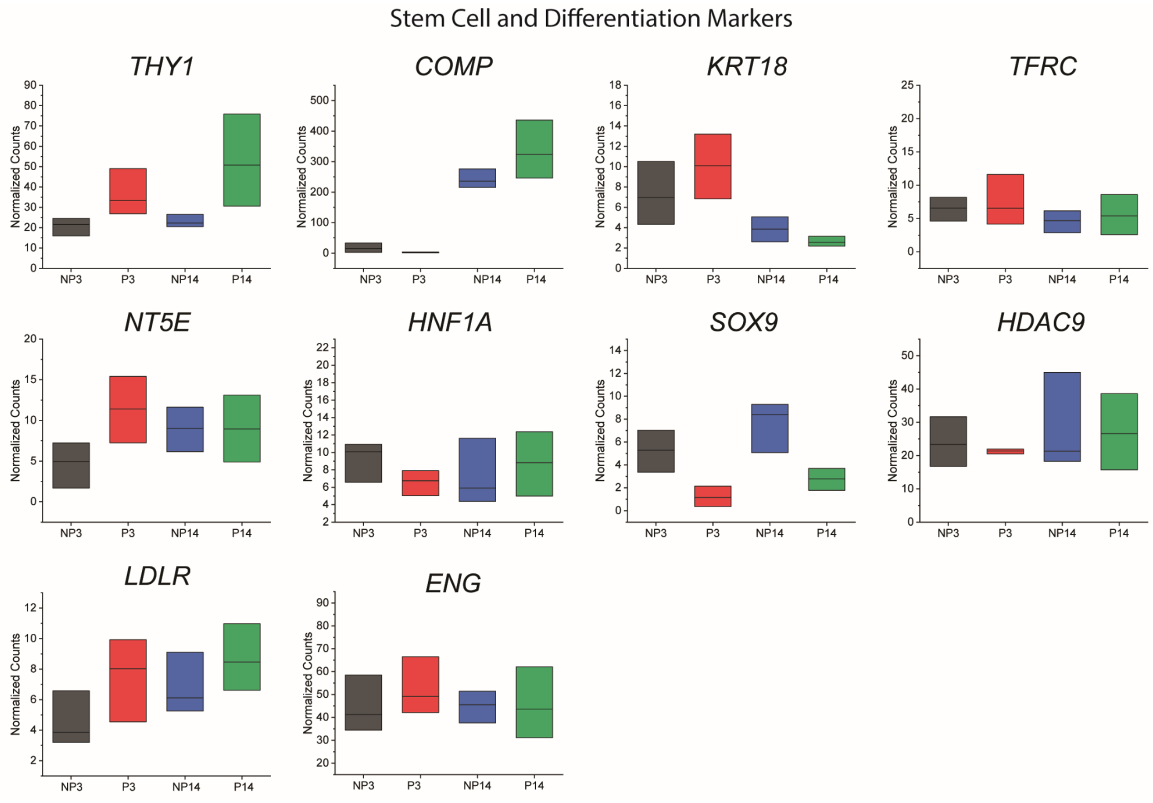
Stem Cell and Differentiation Markers
THY1
COMP
KRT18
TFRC
NT5E
HNF1A
SOX9
HDAC9
LDLR
ENG
Normalized Counts
NP3
P3
NP14
P14

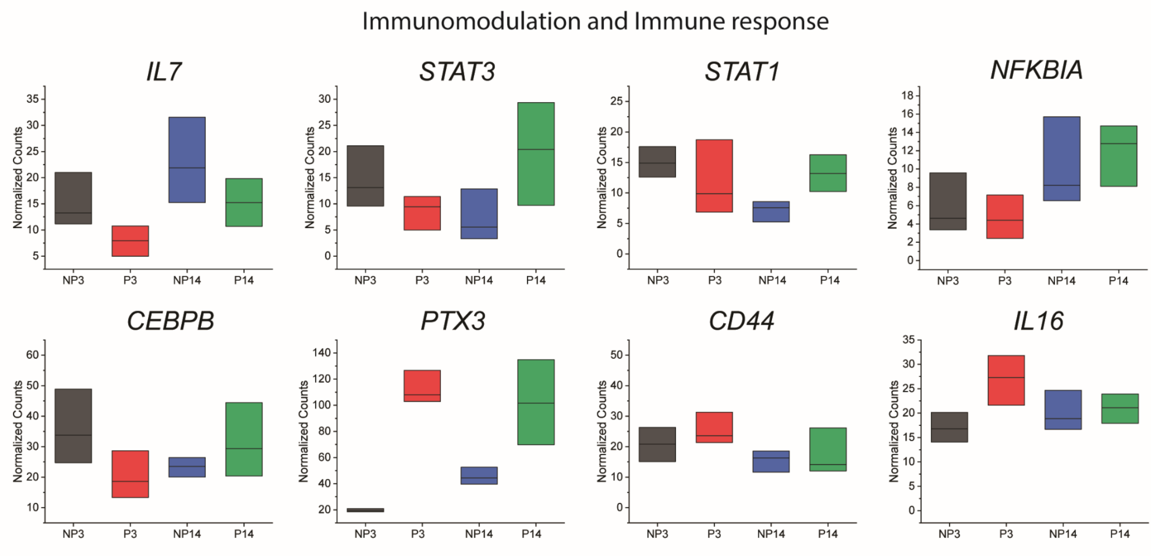
Immunomodulation and Immune response
IL7
STAT3
STAT1
NFKBIA
CEBPB
PTX3
CD44
IL16
Normalized Counts
NP3
P3
NP14
P14

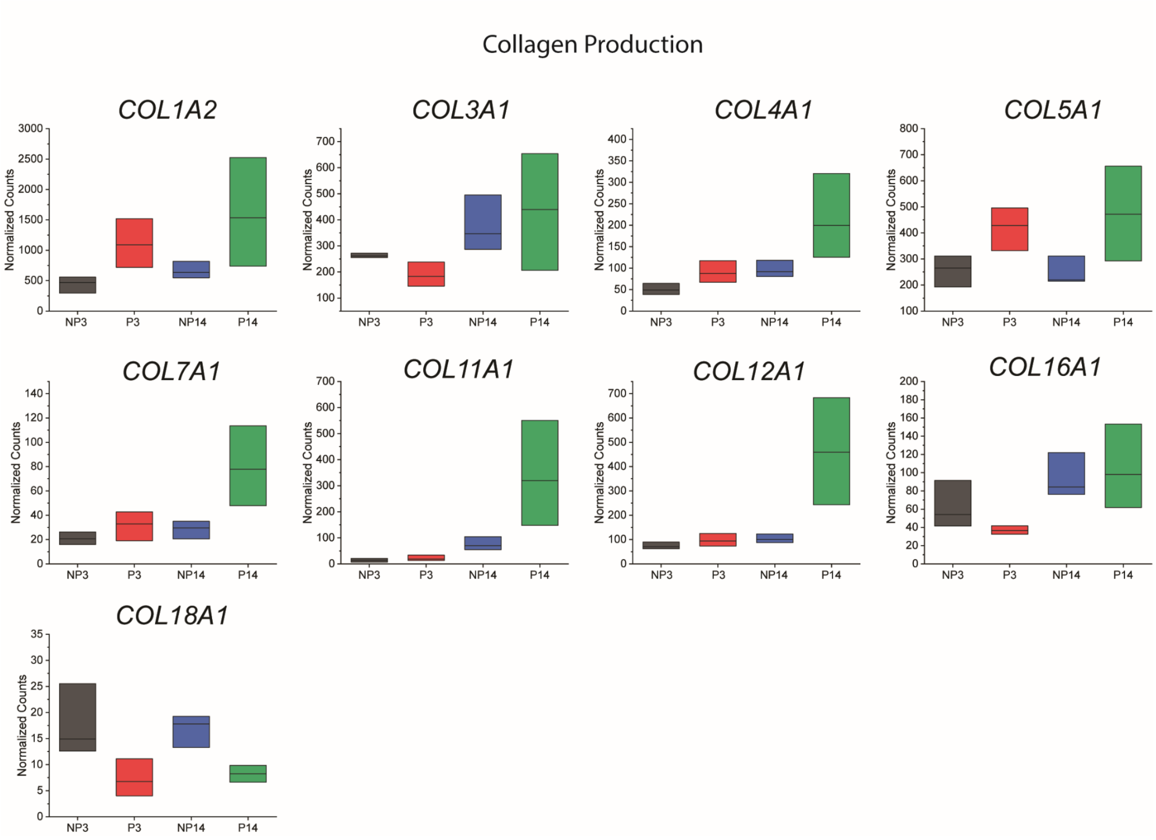

Figure S5. RNA-sequencing data for additional genes of interest. Gene expression is separated into general categories, and graphed as median and interquartile range.

	P3 vs NP3	P3 vs NP3	NP14 VS P3	NP14 VS P3	P14 vs P3	P14 vs P3	P14 vs NP14	P14 vs NP14
Gene	log2FoldChange	padj	log2FoldChange	padj	log2FoldChange	padj	log2FoldChange	padj
COL1A1	1.666206538	0.002476	1.144420999	0.048086489	0.481443083	0.66572578	1.003228622	0.185360809
ALPL	1.248838266	0.270067	1.009989861	0.425390658	0.797397044	0.54287467	1.036245449	0.331276448
IBSP	0	NA	3.603453715	0.024651747	6.36043346	NA	2.236702912	NA
OMD	-0.248909676	NA	2.90542145	0.030728296	3.880627961	NA	0.726296835	NA
SPARC	1.370949027	0.001355	1.464921721	7.26E-05	1.052062744	0.04976294	0.95809005	0.074711612
ITGA5	1.065824092	0.277738	-0.100758871	0.957761614	-0.197710205	0.92199895	0.968872757	0.347458302
VCL	0.646345614	0.329403	0.412649154	0.620973101	0.754763299	0.21729231	0.988459759	0.062721816
PXN	0.089219563	0.947982	-0.159882351	0.874997534	0.128338961	0.90739636	0.377440875	0.57413506
RUNX2	-1.827602696	0.057028	-0.456510589	0.758529114	1.496694616	0.19975033	0.125602508	0.93290031
RHOA	0.774698413	0.262252	0.285587783	0.805142398	0.335332186	0.75637245	0.824442816	0.22448954
MFAP5	0.728567734	0.790481	2.231583487	0.036428445	3.080192012	0.00086112	1.577176259	0.093129737
ENAH	0.475462291	0.444283	0.614690376	0.19223772	0.574689664	0.29942827	0.435461579	0.443251462
ACTB	1.11103572	0.019003	0.195392678	0.855239566	-0.325542963	0.73459214	0.590100079	0.372582132
NEXN	1.216793015	0.019346	1.131379571	0.022311469	0.918501948	0.07904176	1.003915392	0.035668659
CDH2	0.464794647	0.845267	0.369585585	0.867116175	0.275306623	0.90834809	0.370515686	0.825625925
CDH11	-1.039844852	0.108585	0.009096159	0.995790057	1.748078571	0.00234161	0.69913756	0.359948548
GJD3	0.294987447	0.595609	-0.553663023	0.096645776	-0.806620343	0.02097953	0.042030127	0.948895043
ALCAM	1.624236155	0.000363	1.335294329	0.002516581	0.931997898	0.10746585	1.220939724	0.013910591
NOTCH1	0.6540102	NA	0.218804175	0.937738716	0.491811964	NA	0.927017989	NA
FZD4	1.042113993	0.000323	1.070346246	2.87E-05	1.038653499	0.00107583	1.010421246	0.000649247
CTNNB1	-1.014488379	0.112925	-0.200906123	0.872036934	1.483826407	0.01178538	0.67024415	0.363669966
PLOD1	0.869355408	0.378339	0.289389519	0.855239566	0.10972487	0.95515723	0.689690759	0.496167321
LOX	2.064553642	1.56E-08	1.718946862	5.07E-07	0.694770878	0.19063239	1.040377658	0.015603164
MMP2	-1.78101907	7.14E-06	-0.891607936	0.050131976	-0.084619569	0.9506985	-0.9740307	0.078125949
EFEMP1	0.489848722	0.566524	0.672884663	0.264893278	0.812608718	0.15223489	0.629572777	0.29365035
OLFML2A	-0.423919798	0.882713	1.689955567	0.062292846	3.723299794	8.78E-06	1.609424429	0.035854192
LOXL2	2.285819924	0.000239	0.636440287	0.610924259	-0.522263979	0.67572343	1.127115658	0.194233155

LOXL3	0.012250182	0.993555	-0.381843963	0.226674461	-0.723412203	0.01298906	-0.32931806	0.399488435
TPM1	1.81399813	0.002338	1.303557895	0.038537856	0.593235353	0.57900455	1.103675588	0.146599812
MEST	0.653570701	0.512144	0.149185498	0.92528159	0.094560276	0.95515723	0.598945479	0.500296885
PLOD1	0.869355408	0.378339	0.289389519	0.855239566	0.10972487	0.95515723	0.689690759	0.496167321
EBF1	-0.169875114	0.954902	0.540010983	0.763801167	0.945092488	0.52052571	0.235206391	NA
CXCL2	-2.70724846	NA	-2.784523389	NA	1.527993854	NA	1.605268782	NA
ACTA2	0.352080293	0.79015	1.237594404	0.014500687	2.409687601	1.27E-06	1.52417349	0.002983065
CNN2	0.746469867	0.269026	-0.194449017	0.874997534	0.088491009	0.95140155	1.029409893	0.091846893
ENAH	0.475462291	0.444283	0.614690376	0.19223772	0.574689664	0.29942827	0.435461579	0.443251462
PLEC	0.724893018	0.144988	0.491083898	0.408620857	0.85342351	0.0917331	1.08723263	0.012719096
ITGA2	-1.471214099	NA	-0.891999378	0.586968933	-0.20841352	NA	-0.78762824	NA
ITGA3	0.293255369	0.864448	-0.345681604	0.811552986	0.509867602	0.66861623	1.148804575	0.156653797
ITGA7	1.432901419	0.049517	1.981011542	0.000440889	0.490820395	0.65684104	-0.05728973	0.959010455
ITGAV	0.24426255	0.89001	0.363825439	0.781235418	0.803900642	0.35473553	0.684337753	0.420624068
ITGA11	-0.258399002	0.859519	0.40024494	0.6998935	1.723417205	0.00250479	1.064773263	0.10500007
ITGA10	-0.009418179	0.99811	-0.519685161	0.706162569	0.576524097	0.66572578	1.086791079	0.257143745
ITGBL1	-0.022929687	0.99097	0.615391235	0.198003856	1.73165993	5.53E-06	1.093339009	0.006975417
ITGB1	0.389929377	0.501909	0.126067569	0.884192099	0.283893847	0.66697029	0.547755655	0.244009101
ITGB5	-0.319561293	0.807471	-0.248627429	0.832137042	1.096969317	0.09621999	1.026035454	0.116595682
ITGB1BP1	0.527032888	0.245863	0.461194156	0.312936387	0.17517241	0.82191733	0.241011142	0.678259509
SNED1	-0.626496708	0.087724	0.098557575	0.896608774	0.202894587	0.77593082	-0.5221597	0.221139008
TGFB1	-0.300410247	0.749548	-0.418297934	0.539122804	0.526326291	0.41463459	0.644213977	0.254326581
TGFB2	1.611563371	NA	0.841565991	0.791860257	1.055557198	NA	1.825554578	NA
FGF2	-0.304560625	0.754249	0.109481284	0.916317321	-0.41237152	0.6381156	-0.82641343	0.163894634
PDGFA	-0.942160763	NA	0.414822738	NA	2.17132377	NA	0.814340269	NA
PDGFC	-0.594997126	0.769068	-0.432566253	0.815267933	0.652268206	0.70478249	0.489837333	NA
PDGFRA	-0.57002987	0.328382	-0.46534862	0.444479545	0.717929539	0.19028077	0.613248289	0.276348328
PDGFRB	-0.744751924	0.28464	-0.068970708	0.957933143	1.100930237	0.07776202	0.425149022	0.603254647
VEGFC	0.051748807	0.99002	-0.477959839	0.800692804	-0.066745058	0.97795466	0.462963587	0.762828223
IGF2	-1.075838751	0.031207	0.601673408	0.251293599	3.163661315	6.68E-17	1.486149156	1.60E-05
EGFR	-0.671417384	0.604066	-0.759263988	0.472570786	0.394714035	0.79973675	0.48256064	0.692185143

FGF7	1.53151601	0.004787	2.414332283	9.36E-09	2.649957714	5.20E-14	1.767141441	8.26E-09
BMP1	0.486637821	0.714432	0.432782649	0.718871599	0.505826501	0.65247916	0.559681674	0.547035183
BMP6	0.528663616	0.051572	0.326589509	0.330613489	-0.49940797	0.11232057	-0.29733386	0.422504655
BMP8A	0.092866636	0.959187	0.617735854	0.422261613	-0.011481715	0.99260167	-0.53635093	0.511432865
THY1	0.494730185	0.660986	-0.032155952	0.984519433	0.503621168	0.62011711	1.030507305	0.136662188
COMP	-3.274263044	1.18E-06	3.27973852	8.31E-23	7.11174054	5.71E-32	0.557738976	0.347447469
KRT18	0.42024024	0.799509	-0.877585797	0.424553227	-1.956297447	0.04731444	-0.65847141	0.668564231
TFRC	0.013736879	0.99811	-0.772885824	0.607008949	-0.458819627	0.8162231	0.327803076	0.850157747
NT5E	0.597671069	0.727284	0.289440975	0.883873812	-0.231453976	0.91441687	0.076776118	0.962094551
HNF1A	-0.553340189	0.697072	-0.282685993	0.851840459	0.38426435	0.79813493	0.113610155	0.931878025
SOX9	-2.063627274	0.061134	0.348610896	0.836336495	0.949137795	0.62557171	-1.46310037	0.212004538
HDAC9	-0.097949605	0.960047	0.311884765	0.811552986	0.33146642	0.79973675	-0.07836795	0.948729818
LDLR	0.254262666	0.909341	0.143664686	0.941246323	0.382226179	0.81497467	0.492824158	0.69048677
ENG	0.104826627	0.943984	-0.038956685	0.97356656	-0.162029869	0.8924509	-0.01824656	0.987787675
IL7	-0.993138536	0.16442	0.559116575	0.486577972	0.964196602	0.21756549	-0.58805851	0.460305481
STAT3	-0.811619197	0.572654	-0.928136585	0.427356174	1.278792611	0.2384136	1.395309999	0.165836989
STAT1	-0.341298467	0.817966	-0.937690826	0.19757614	0.198028345	0.89777816	0.794420704	0.363790959
NFKBIA	-0.355890058	0.875675	0.728764445	0.574634954	1.237844529	0.25334286	0.153190026	0.914881727
CEBPB	-1.057010645	0.015783	-0.776329385	0.075427407	0.697549333	0.24776885	0.416868072	0.527708964
PTX3	2.407754051	2.70E-14	1.133637729	0.001108069	-0.143961344	0.87325326	1.130154978	0.001660566
CD44	0.114639584	0.954569	-0.584395512	0.574824867	-0.368953566	0.7862982	0.33008153	0.76796823
IL16	0.623882934	0.253014	0.292333979	0.723469118	-0.35916945	0.63914758	-0.02762049	0.976018633
COL1A2	1.077765751	0.238037	0.551212448	0.669110685	0.545406586	0.69578042	1.071959889	0.26902418
COL3A1	-0.54140954	0.637243	0.524726466	0.599264926	1.166927185	0.11547893	0.100791179	0.931726605
COL4A1	0.509620267	0.595611	0.760488534	0.236547826	1.288100788	0.02943558	1.037232521	0.095187554
COL5A1	0.549786739	0.335337	-0.09957021	0.921966835	0.202312778	0.82997232	0.851669726	0.079878031
COL7A1	0.500340721	0.652508	0.568919433	0.522548982	1.421736849	0.01610711	1.353158136	0.017076501
COL11A1	0.633866734	0.698247	2.470806822	5.76E-05	3.87319527	1.29E-08	2.036255182	0.006271043
COL12A1	0.294987013	0.786464	0.534905984	0.422038383	2.223354799	1.09E-07	1.983435828	4.07E-07
COL16A1	-0.692794289	0.329403	0.817320056	0.13899287	1.55663799	0.00303135	0.046523645	0.962373455
COL18A1	-1.224245908	0.045228	-0.200601136	0.870908961	0.027276178	0.98825473	-0.99636859	0.19138024

Figure S6. Log2 fold change and adjusted p-value for comparisons between groups of all graphed individual genes. Log2 fold change > 2 and adjusted p-value < 0.05 are highlighted in blue.

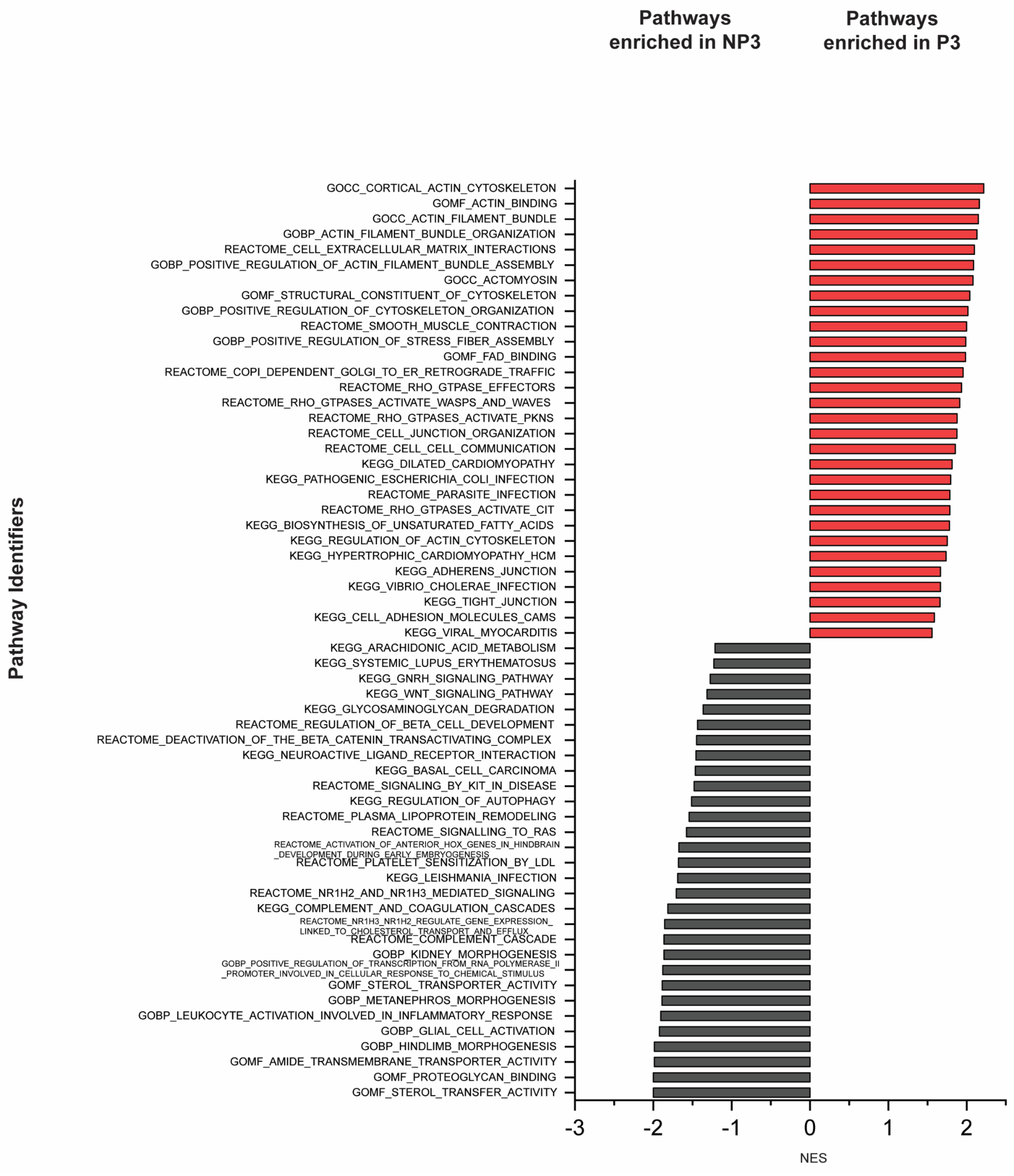
Pathways enriched in NP3
Pathways enriched in P3
Pathway Identifiers
GOCC_CORTICAL_ACTIN_CYTOSKELETON
GOMF_ACTIN_BINDING
GOCC_ACTIN_FILAMENT_BUNDLE
GOBP_ACTIN_FILAMENT_BUNDLE_ORGANIZATION
REACTOME_CELL_EXTRACELLULAR_MATRIX_INTERACTIONS
GOBP_POSITIVE_REGULATION_OF_ACTIN_FILAMENT_BUNDLE_ASSEMBLY
GOCC_ACTOMYOSIN
GOMF_STRUCTURAL_CONSTITUENT_OF_CYTOSKELETON
GOBP_POSITIVE_REGULATION_OF_CYTOSKELETON_ORGANIZATION
REACTOME_SMOOTH_MUSCLE_CONTRACTION
GOBP_POSITIVE_REGULATION_OF_STRESS_FIBER_ASSEMBLY
GOMF_FAD_BINDING
REACTOME_COPI_DEPENDENT_GOLGI_TO_ER_RETROGRADE_TRAFFIC
REACTOME_RHO_GTPASE_EFFECTORS
REACTOME_RHO_GTPASES_ACTIVATE_WASPS_AND_WAVES
REACTOME_RHO_GTPASES_ACTIVATE_PKNS
REACTOME_CELL_JUNCTION_ORGANIZATION
REACTOME_CELL_CELL_COMMUNICATION
KEGG_DILATED_CARDIOMYOPATHY
KEGG_PATHOGENIC_ESCHERICHIA_COLI_INFECTION
REACTOME_PARASITE_INFECTION
REACTOME_RHO_GTPASES_ACTIVATE_CIT
KEGG_BIOSYNTHESIS_OF_UNSATURATED_FATTY_ACIDS
KEGG_REGULATION_OF_ACTIN_CYTOSKELETON
KEGG_HYPERTROPHIC_CARDIOMYOPATHY_HCM
KEGG_ADHERENS_JUNCTION
KEGG_VIBRIO_CHOLERAE_INFECTION
KEGG_TIGHT_JUNCTION
KEGG_CELL_ADHESION_MOLECULES_CAMS
KEGG_VIRAL_MYOCARDITIS
KEGG_ARACHIDONIC_ACID_METABOLISM
KEGG_SYSTEMIC_LUPUS_ERYTHEMATOSUS
KEGG_GNRH_SIGNALING_PATHWAY
KEGG_WNT_SIGNALING_PATHWAY
KEGG_GLYCOSAMINOGLYCAN_DEGRADATION
REACTOME_REGULATION_OF_BETA_CELL_DEVELOPMENT
REACTOME_DEACTIVATION_OF_THE_BETA_CATENIN_TRANSACTIVATING_COMPLEX
KEGG_NEUROACTIVE_LIGAND_RECEPTOR_INTERACTION
KEGG_BASAL_CELL_CARCINOMA
REACTOME_SIGNALING_BY_KIT_IN_DISEASE
KEGG_REGULATION_OF_AUTOPHAGY
REACTOME_PLASMA_LIPOPROTEIN_REMODELING
REACTOME_SIGNALLING_TO_RAS
REACTOME_ACTIVATION_OF_ANTERIOR_HOX_GENES_IN_HINDBRAIN
_DEVELOPMENT_DURING_EARLY_EMBRYOGENESIS
REACTOME_PLATELET_SENSITIZATION_BY_LDL
KEGG_LEISHMANIA_INFECTION
REACTOME_NR1H2_AND_NR1H3_MEDIATED_SIGNALING
KEGG_COMPLEMENT_AND_COAGULATION_CASCADES
REACTOME_NR1H3_NR1H2_REGULATE_GENE_EXPRESSION_
LINKED_TO_CHOLESTEROL_TRANSPORT_AND_EFFLUX
REACTOME_COMPLEMENT_CASCADE
GOBP_KIDNEY_MORPHOGENESIS
GOBP_POSITIVE_REGULATION_OF_TRANSCRIPTION_FROM_RNA_POLYMERASE_II
_PROMOTER_INVOLVED_IN_CELLULAR_RESPONSE_TO_CHEMICAL_STIMULUS
GOMF_STEROL_TRANSPORTER_ACTIVITY
GOBP_METANEPHROS_MORPHOGENESIS
GOBP_LEUKOCYTE_ACTIVATION_INVOLVED_IN_INFLAMMATORY_RESPONSE
GOBP_GLIAL_CELL_ACTIVATION
GOBP_HINDLIMB_MORPHOGENESIS
GOMF_AMIDE_TRANSMEMBRANE_TRANSPORTER_ACTIVITY
GOMF_PROTEOGLYCAN_BINDING
GOMF_STEROL_TRANSFER_ACTIVITY
-3
-2
-1
0
1
2
NES

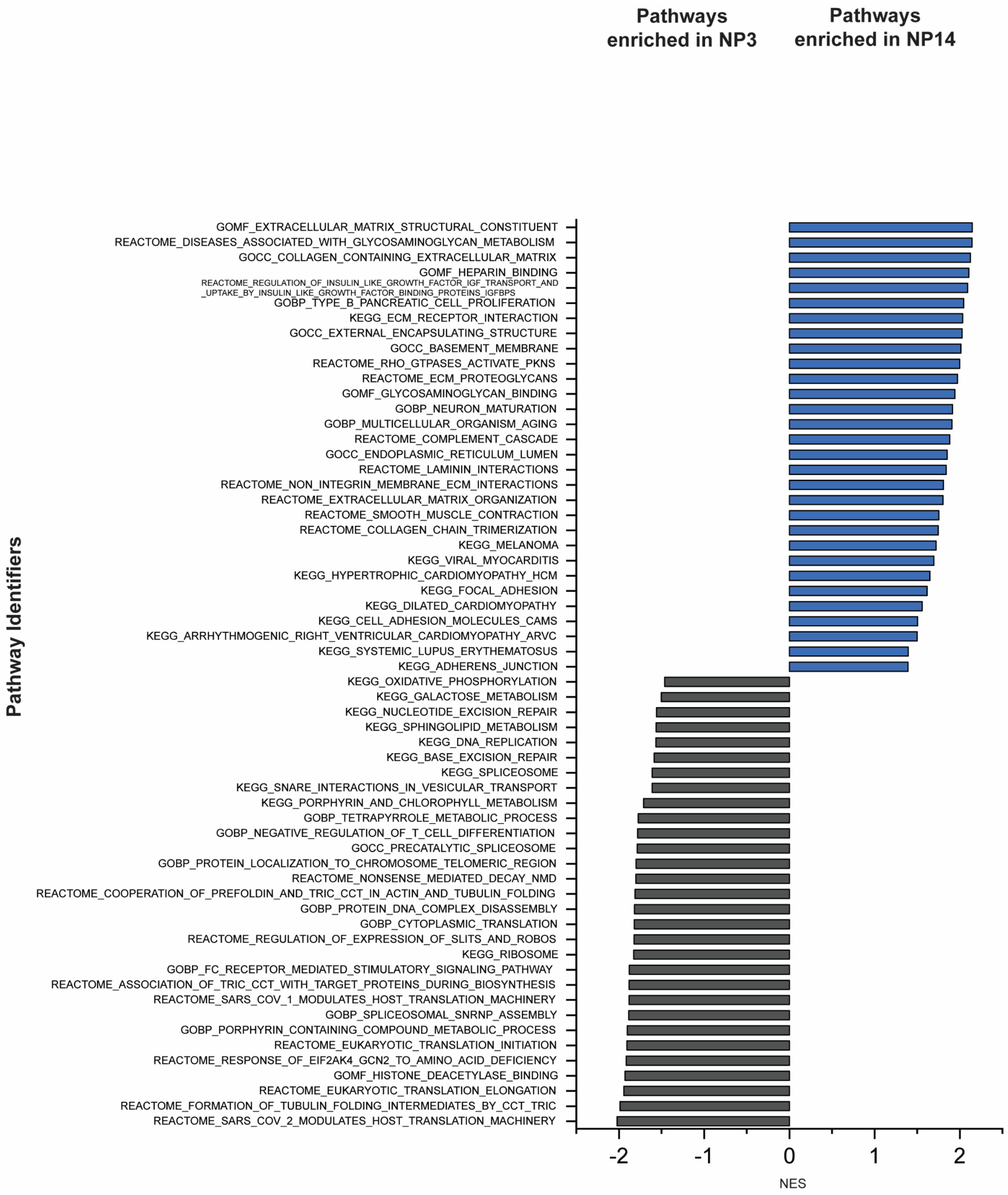

Pathways enriched in NP3
Pathways enriched in NP14
Pathway Identifiers
GOMF_EXTRACELLULAR_MATRIX_STRUCTURAL_CONSTITUENT
REACTOME_DISEASES_ASSOCIATED_WITH_GLYCOSAMINOGLYCAN_METABOLISM
GOCC_COLLAGEN_CONTAINING_EXTRACELLULAR_MATRIX
GOMF_HEPARIN_BINDING
REACTOME_REGULATION_OF_INSULIN_LIKE_GROWTH_FACTOR_IGF_TRANSPORT_AND
_UPTAKE_BY_INSULIN_LIKE_GROWTH_FACTOR_BINDING_PROTEINS_IGFBPS
GOBP_TYPE_B_PANCREATIC_CELL_PROLIFERATION
KEGG_ECM_RECEPTOR_INTERACTION
GOCC_EXTERNAL_ENCAPSULATING_STRUCTURE
GOCC_BASEMENT_MEMBRANE
REACTOME_RHO_GTPASES_ACTIVATE_PKNS
REACTOME_ECM_PROTEOGLYCANS
GOMF_GLYCOSAMINOGLYCAN_BINDING
GOBP_NEURON_MATURATION
GOBP_MULTICELLULAR_ORGANISM_AGING
REACTOME_COMPLEMENT_CASCADE
GOCC_ENDOPLASMIC_RETICULUM_LUMEN
REACTOME_LAMININ_INTERACTIONS
REACTOME_NON_INTEGRIN_MEMBRANE_ECM_INTERACTIONS
REACTOME_EXTRACELLULAR_MATRIX_ORGANIZATION
REACTOME_SMOOTH_MUSCLE_CONTRACTION
REACTOME_COLLAGEN_CHAIN_TRIMERIZATION
KEGG_MELANOMA
KEGG_VIRAL_MYOCARDITIS
KEGG_HYPERTROPHIC_CARDIOMYOPATHY_HCM
KEGG_FOCAL_ADHESION
KEGG_DILATED_CARDIOMYOPATHY
KEGG_CELL_ADHESION_MOLECULES_CAMS
KEGG_ARRHYTHMOGENIC_RIGHT_VENTRICULAR_CARDIOMYOPATHY_ARVC
KEGG_SYSTEMIC_LUPUS_ERYTHEMATOSUS
KEGG_ADHERENS_JUNCTION
KEGG_OXIDATIVE_PHOSPHORYLATION
KEGG_GALACTOSE_METABOLISM
KEGG_NUCLEOTIDE_EXCISION_REPAIR
KEGG_SPHINGOLIPID_METABOLISM
KEGG_DNA_REPLICATION
KEGG_BASE_EXCISION_REPAIR
KEGG_SPLICEOSOME
KEGG_SNARE_INTERACTIONS_IN_VESICULAR_TRANSPORT
KEGG_PORPHYRIN_AND_CHLOROPHYLL_METABOLISM
GOBP_TETRAPYRROLE_METABOLIC_PROCESS
GOBP_NEGATIVE_REGULATION_OF_T_CELL_DIFFERENTIATION
GOCC_PRECATALYTIC_SPLICEOSOME
GOBP_PROTEIN_LOCALIZATION_TO_CHROMOSOME_TELOMERIC_REGION
REACTOME_NONSENSE_MEDIATED_DECAY_NMD
REACTOME_COOPERATION_OF_PREFOLDIN_AND_TRIC_CCT_IN_ACTIN_AND_TUBULIN_FOLDING
GOBP_PROTEIN_DNA_COMPLEX_DISASSEMBLY
GOBP_CYTOPLASMIC_TRANSLATION
REACTOME_REGULATION_OF_EXPRESSION_OF_SLITS_AND_ROBOS
KEGG_RIBOSOME
GOBP_FC_RECEPTOR_MEDIATED_STIMULATORY_SIGNALING_PATHWAY
REACTOME_ASSOCIATION_OF_TRIC_CCT_WITH_TARGET_PROTEINS_DURING_BIOSYNTHESIS
REACTOME_SARS_COV_1_MODULATES_HOST_TRANSLATION_MACHINERY
GOBP_SPLICEOSOMAL_SNRNP_ASSEMBLY
GOBP_PORPHYRIN_CONTAINING_COMPOUND_METABOLIC_PROCESS
REACTOME_EUKARYOTIC_TRANSLATION_INITIATION
REACTOME_RESPONSE_OF_EIF2AK4_GCN2_TO_AMINO_ACID_DEFICIENCY
GOMF_HISTONE_DEACETYLASE_BINDING
REACTOME_EUKARYOTIC_TRANSLATION_ELONGATION
REACTOME_FORMATION_OF_TUBULIN_FOLDING_INTERMEDIATES_BY_CCT_TRIC
REACTOME_SARS_COV_2_MODULATES_HOST_TRANSLATION_MACHINERY
-2
-1
0
1
2
NES

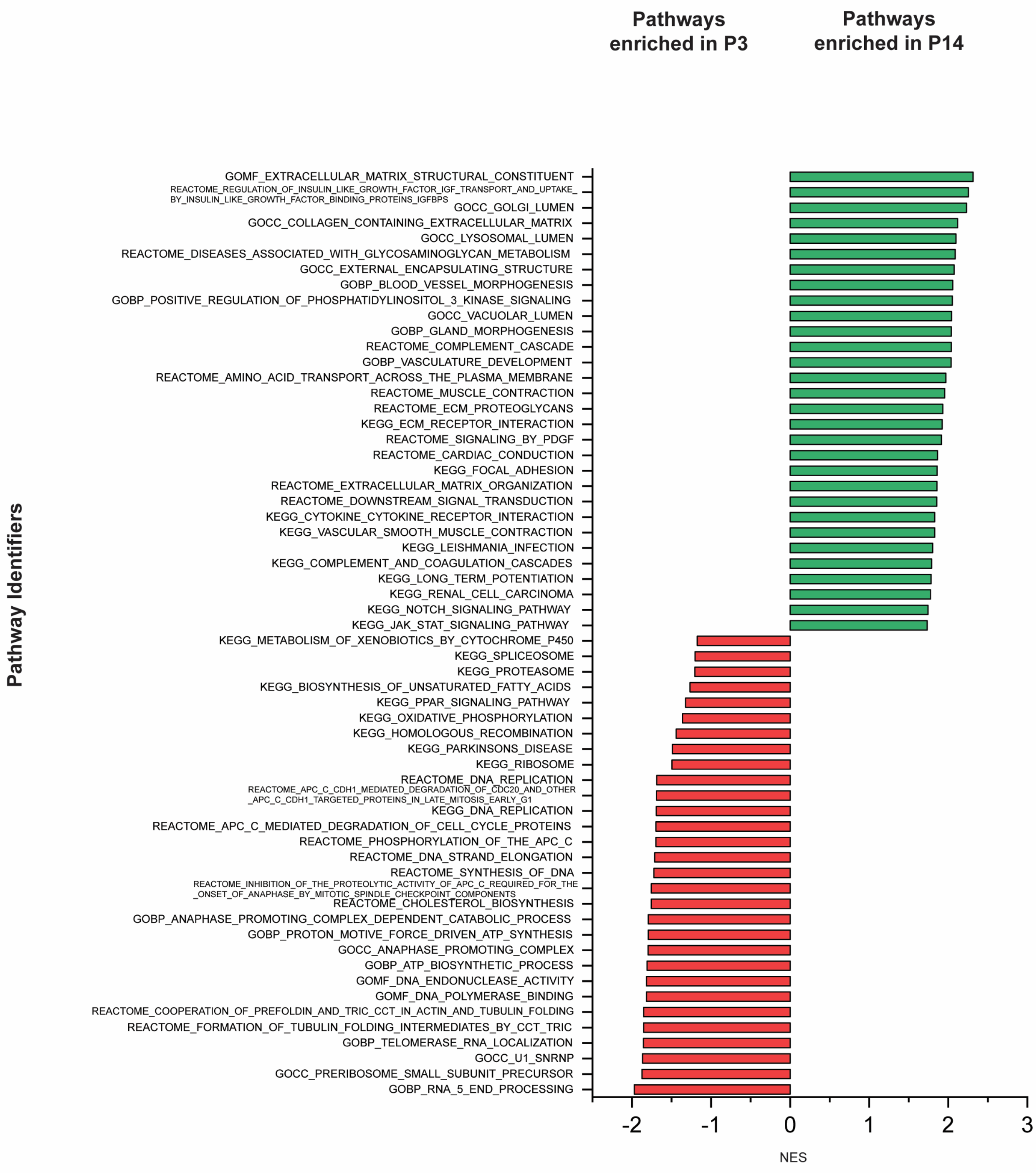
Pathways enriched in P3
Pathways enriched in P14
Pathway Identifiers
GOMF_EXTRACELLULAR_MATRIX_STRUCTURAL_CONSTITUENT
REACTOME_REGULATION_OF_INSULIN_LIKE_GROWTH_FACTOR_IGF_TRANSPORT_AND_UPTAKE_BY_INSULIN_LIKE_GROWTH_FACTOR_BINDING_PROTEINS_IGFBPS
GOCC_GOLGI_LUMEN
GOCC_COLLAGEN_CONTAINING_EXTRACELLULAR_MATRIX
GOCC_LYSOSOMAL_LUMEN
REACTOME_DISEASES_ASSOCIATED_WITH_GLYCOSAMINOGLYCAN_METABOLISM
GOCC_EXTERNAL_ENCAPSULATING_STRUCTURE
GOBP_BLOOD_VESSEL_MORPHOGENESIS
GOBP_POSITIVE_REGULATION_OF_PHOSPHATIDYLINOSITOL_3_KINASE_SIGNALING
GOCC_VACUOLAR_LUMEN
GOBP_GLAND_MORPHOGENESIS
REACTOME_COMPLEMENT_CASCADE
GOBP_VASCULATURE_DEVELOPMENT
REACTOME_AMINO_ACID_TRANSPORT_ACROSS_THE_PLASMA_MEMBRANE
REACTOME_MUSCLE_CONTRACTION
REACTOME_ECM_PROTEOGLYCANS
KEGG_ECM_RECEPTOR_INTERACTION
REACTOME_SIGNALING_BY_PDGF
REACTOME_CARDIAC_CONDUCTION
KEGG_FOCAL_ADHESION
REACTOME_EXTRACELLULAR_MATRIX_ORGANIZATION
REACTOME_DOWNSTREAM_SIGNAL_TRANSDUCTION
KEGG_CYTOKINE_CYTOKINE_RECEPTOR_INTERACTION
KEGG_VASCULAR_SMOOTH_MUSCLE_CONTRACTION
KEGG_LEISHMANIA_INFECTION
KEGG_COMPLEMENT_AND_COAGULATION_CASCADES
KEGG_LONG_TERM_POTENTIATION
KEGG_RENAL_CELL_CARCINOMA
KEGG_NOTCH_SIGNALING_PATHWAY
KEGG_JAK_STAT_SIGNALING_PATHWAY
KEGG_METABOLISM_OF_XENOBIOTICS_BY_CYTOCHROME_P450
KEGG_SPLICEOSOME
KEGG_PROTEASOME
KEGG_BIOSYNTHESIS_OF_UNSATURATED_FATTY_ACIDS
KEGG_PPAR_SIGNALING_PATHWAY
KEGG_OXIDATIVE_PHOSPHORYLATION
KEGG_HOMOLOGOUS_RECOMBINATION
KEGG_PARKINSONS_DISEASE
KEGG_RIBOSOME
REACTOME_DNA_REPLICATION
REACTOME_APC_C_CDH1_MEDIATED_DEGRADATION_OF_CDC20_AND_OTHER_APC_C_CDH1_TARGETED_PROTEINS_IN_LATE_MITOSIS_EARLY_G1
KEGG_DNA_REPLICATION
REACTOME_APC_C_MEDIATED_DEGRADATION_OF_CELL_CYCLE_PROTEINS
REACTOME_PHOSPHORYLATION_OF_THE_APC_C
REACTOME_DNA_STRAND_ELONGATION
REACTOME_SYNTHESIS_OF_DNA
REACTOME_INHIBITION_OF_THE_PROTEOLYTIC_ACTIVITY_OF_APC_C_REQUIRED_FOR_THE_ONSET_OF_ANAPHASE_BY_MITOTIC_SPINDLE_CHECKPOINT_COMPONENTS
REACTOME_CHOLESTEROL_BIOSYNTHESIS
GOBP_ANAPHASE_PROMOTING_COMPLEX_DEPENDENT_CATABOLIC_PROCESS
GOBP_PROTON_MOTIVE_FORCE_DRIVEN_ATP_SYNTHESIS
GOCC_ANAPHASE_PROMOTING_COMPLEX
GOBP_ATP_BIOSYNTHETIC_PROCESS
GOMF_DNA_ENDONUCLEASE_ACTIVITY
GOMF_DNA_POLYMERASE_BINDING
REACTOME_COOPERATION_OF_PREFOLDIN_AND_TRIC_CCT_IN_ACTIN_AND_TUBULIN_FOLDING
REACTOME_FORMATION_OF_TUBULIN_FOLDING_INTERMEDIATES_BY_CCT_TRIC
GOBP_TELOMERASE_RNA_LOCALIZATION
GOCC_U1_SNRNP
GOCC_PRERIBOSOME_SMALL_SUBUNIT_PRECURSOR
GOBP_RNA_5_END_PROCESSING
-2
-1
0
1
2
3
NES

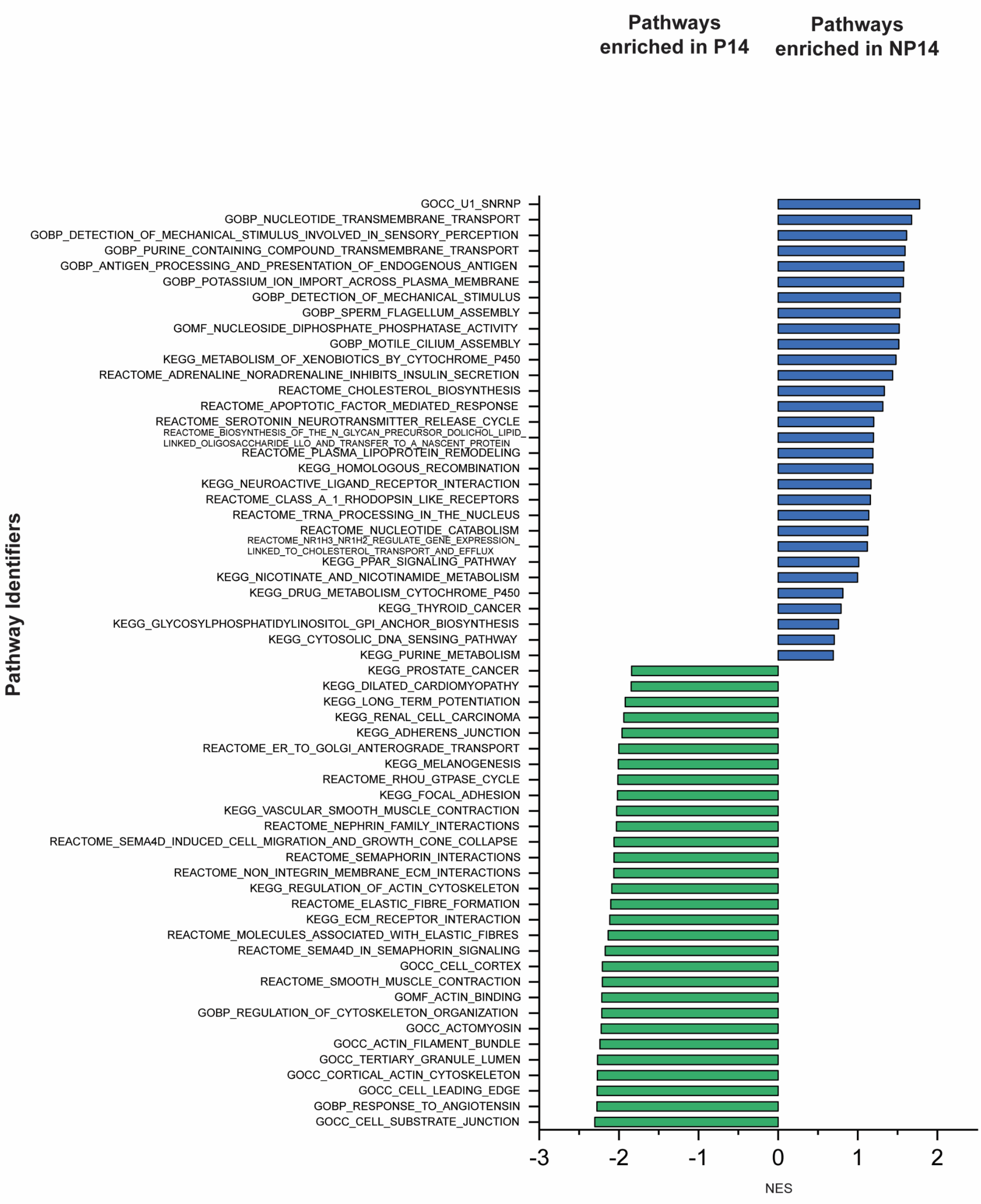

Figure S7. Gene set enrichment analysis top pathways sorted by NES for each pairwise comparison between sample conditions.
